# Occupational physical activity, mortality and CHD events in the Italian Longitudinal Study

**DOI:** 10.1007/s00420-021-01765-0

**Published:** 2021-10-11

**Authors:** Elena Strippoli, Amanda Hughes, Gabriella Sebastiani, Paola Di Filippo, Angelo d’Errico

**Affiliations:** 1 Epidemiology Department, Local Health Unit TO3, Piedmont Region, Grugliasco, Torino, Italy; 2 MRC Integrative Epidemiology Unit, University of Bristol, Bristol, BS8 2BN UK; 3 National Institute of Statistics (ISTAT), Rome, Italy

**Keywords:** Occupational health, Physical activity, Health inequalities, Mortality, CHD

## Abstract

**Purpose:**

Several recent studies have suggested a ‘physical activity paradox’ whereby leisure-time physical activity benefits health, but occupational physical activity is harmful. However, other studies imply that occupational physical activity is beneficial. Using data from a nationally representative Italian sample, we investigate if the context, or domain, of physical activity matters for mortality and coronary heart disease (CHD) events.

**Methods:**

Among 40,220 men and women aged 40–55 at baseline, we used Cox models to compare associations of occupational, domestic and leisure-time physical activity with risk of mortality and CHD events over a follow-up period of up to 14 years. We accounted for sociodemographic factors, smoking, body mass index (BMI), physical and mental health, and educational qualifications.

**Results:**

Occupational physical activity was not significantly associated with risk of mortality or CHD events for women, or with CHD events for men. In crude models, risk of mortality was higher for men in the highest occupational activity group, compared to the lowest (HR 1.26, 95% CI 1.01, 1.57). This attenuated with adjustment for health-related behaviours, health, and education (HR 1.03, 95% CI 0.77, 1.38). In crude models, leisure-time physical activity was significantly associated with decreased mortality and CHD risk only for men. Domestic physical activity was not associated with either outcome for either gender.

**Conclusion:**

In a large sample of middle-aged Italian workers, we found limited evidence of harmful or beneficial effects of occupational physical activity on mortality or CHD events. However, confidence intervals were wide, and results consistent with a range of effects in both directions.

**Supplementary Information:**

The online version contains supplementary material available at 10.1007/s00420-021-01765-0.

## Introduction

The World Health Organization (WHO) guidelines on physical activity state that activity ‘at work, leisure, home or during transportation’ all count towards recommended weekly amounts (Bull et al. 2020). However, while leisure-time physical activity (LTPA) has been consistently found to be associated with good health (Cheng et al. 2018), occupational physical activity (OPA) has not. Early studies reported positive associations of OPA with health, suggesting that physical activity promotes health regardless of the context or domain (Samitz et al. 2011). However, a 2018 meta-analysis concluded that high levels of occupational physical activity were associated with increased risk of early death, at least among men (Coenen et al. 2018). This discrepancy, according to which the domain of physical activity matters for associations, has been called the ‘physical activity paradox’ (Holtermann et al. 2018). One explanation is that physical activity at work differs in important respects from leisure-time physical activity (Hallman et al. 2017). In contrast to LTPA, intensity levels of OPA are often insufficient to improve cardiorespiratory fitness. At the same time, OPA often involves heavy lifting and awkward postures, and is performed over long periods with insufficient recovery time. Consequently, biological effects of OPA could be harmful rather than beneficial, especially for workers with pre-existing poor health (Holtermann et al. 2018). Alternatively, the apparent paradox could reflect different sources of confounding. LTPA may be more common among individuals with better pre-existing health or more advantaged socioeconomic position. Underlying health and socioeconomic advantage are difficult to fully account for, and residual confounding may inflate apparent benefits of LTPA. Meanwhile, occupational physical activity is more common in lower-paid and lower-status jobs, and associations of OPA with worse health may therefore reflect confounding by factors including income (von dem Knesebeck et al. 2017), or specific job-related psychosocial exposures, such as demand-control or effort-reward imbalance (Reinhardt et al. 2013), or job insecurity (Ferrie 2001). Confounding by health-related behaviours such as diet and smoking, more prevalent in manual occupational social class groups (Windsor-Shellard et al. 2020), where more intense OPA is likely to be common, may also contribute to an apparent physical activity paradox (Shephard 2019). Studies of OPA and mortality based on administrative data are often unable to take smoking or BMI into account, including two recent Finnish studies. One of these supported the physical activity paradox for men (Mikkola et al. 2019), while the other found that premature mortality was highest for men in the third OPA quartile (Ervasti et al. 2019). Neither supported an effect for women. Since the 2018 meta-analysis (Coenen et al. 2018) included studies which adjusted only for age, gender, and one other social or health-related factor, pooled results may reflect the influence of factors not taken into account. Moreover, residual confounding may affect estimates even where relevant covariates are included (Shephard 2019). While confounding by socioeconomic factors and health-related behaviours would likely inflate negative health effects of OPA, health-related selection may lead to harmful effects of OPA being underestimated. Individuals in poor health may be more likely to retire early from physically demanding jobs, and thus not be observed in studies. Secondly, employees in poorer health may be transferred to less active tasks within the workplace (Flower et al. 2019), leading to misclassification of exposure. Both processes would be expected to bias negative health effects of OPA towards the null.

In this context, it is perhaps unsurprising that studies have produced heterogeneous results. The 2018 meta-analysis incorporated 16 studies, of which five reported a detrimental association, five reported a protective association, and six reported no association (Coenen et al. 2018). More recent results are equally mixed. In the past year, a large study of Danish men and women (*N* = 104,046) reported a robust association of OPA with increased risk of mortality and cardiovascular events, after accounting for a wide range of socioeconomic factors, health-related behaviours, and biomarker-based measures of health (Holtermann et al. 2021). These results contrast with the results of another large study, published the same year, of OPA and longevity in 437,378 men and women from Norway (Dalene et al. 2021). Here, no clear associations were seen for women, while for men, higher OPA was in fully adjusted models associated with increased longevity. Many studies have reported less evidence of a physical activity paradox for women than men (Coenen et al. 2018; Ervasti et al. 2019; Mikkola et al. 2019; Wanner et al. 2019), with several explanations proposed. Firstly, physically demanding work may be more common among men than women, or differ in its nature (Hallman et al. 2015). It has also been suggested that benefits and risks associated with particular types of physical activity may differ between men and women for physiological reasons (Hands et al. 2016). Thirdly, occupational physical activity is usually measured via self-report, so gender differences in reporting could account for different associations with health outcomes (Coenen et al. 2018). Lastly, this may point to gender differences not in causal effects of OPA, but rather in a complex network of confounding factors influencing associations (Dalene et al. 2021).

While the 2020 WHO guidelines do count OPA towards recommended amounts of physical activity, the Guideline Development Group also concluded that ‘there was insufficient evidence to determine whether specific health benefits vary by type or domain of physical activity’(Bull et al. 2020). Strengthening the evidence base on the health effects of OPA is therefore critical. We investigate the relationship of OPA, LTPA and DPA with all-cause mortality and with CHD events, using data from follow-up of two nationally representative Italian surveys from 1999 to 2000 and 2004 to 2005. This is the first investigation of this question in Italy using national data. We adjust for key socio-demographic factors, underlying health, and health-related behaviours, and use a directly reported rather than predicted measure of OPA. To minimise bias due to health-related selection, including the impact of early retirement and the transfer of older workers to less active tasks, we restrict analyses to adults aged 40–55.

## Methods

### Population

We used data from The Italian Longitudinal Study, a follow-up achieved through record linkage of the 1999–2000 and 2004–2005 Italian National Health Interview Surveys (NHIS) with the Italian National Archives of Causes of Death (from the Italian National Institute of Statistics (ISTAT)) and hospital discharge records (Ministry of Health). The NHIS is a representative survey of the Italian population conducted approximately every 5 years by the Italian National Institute of Statistics (hereafter ISTAT). It has a two-stage sampling design, with municipalities as primary sampling units and households as secondary units Marinacci et al. (2013). The NHIS provide information on health and disability, overweight and obesity, health-related behaviours (e.g., physical activity, smoking) and use of health services, as well as information on individual and household socioeconomic characteristics. The questionnaire is administered through a personal interview, except for a self-completion part which asks about chronic morbidities, smoking, perceived health status, pregnancy and childbirth, medication use, and views about health services.

The NHIS was conducted on representative samples of the resident population in Italy. It surveyed 52,332 families and 140,011 individuals for the 1999–2000 survey, and 50,474 families and 128,040 individuals for the 2004–2005 survey. NHIS data were linked to mortality and admissions data using the fiscal code. This is an alphanumeric code based on first and last name, date and place of birth, and gender, which uniquely identified each Italian citizen. This is used for fiscal purposes and in relation to accessing public services, including health services. The information necessary to construct the fiscal codes was available for 92% of NHIS 1999–2000 interviewees (128,818) and 98.3% of NHIS 2004–2005 interviewees (125,850). Participants were followed up for mortality and hospitalization from baseline until 2014. Previous analysis has shown that linked and unlinked individuals did not differ significantly for the main sociodemographic characteristics and health status variables. The overall number of deaths observed corresponded to about 90% of the expected deaths based on Italian mortality rates for the whole period (90.5% for the 1999–2000 follow-up and 89.9% for the 2004–2005 follow-up). For hospital discharge, the proportions between observed and expected discharges were 85.3% for the 1999–2000 follow-up and 88.9% for the 2004–2005 follow-up. Further details are available elsewhere (Sebastiani et al. 2019).

The present analysis was restricted to participants who at baseline were aged 40–55 and currently in work (*N* = 40,220 across both surveys). We restricted to this age group to reduce the influence of health-related selection into retirement. At the time of the surveys, the statutory pension age in Italy was 60 for women and 65 men, but it was nevertheless possible for individuals who had worked for 35 years to access a pension at age 55 in 2000 and at age 57 in 2005. Thus, many manual workers of this generation who began working before age 20 would have retired before age 60. Only currently working participants were included because the surveys did not ask about physical activity in former employment. In the 1999–2000 survey, it was not possible to distinguish full-time from part-time workers, so we include workers regardless of weekly hours. Two outcomes were considered: all-cause mortality, and CHD events (death or hospitalization) identified as ICD-9 codes 410–414 or ICD-10 codes I20-I25. Between baseline interview and the end of follow-up, there were 1430 deaths and 1649 CHD events (Supplementary Table S3).

### Occupational, domestic and leisure-time physical activity

Appendix A shows the survey questions relating to physical activity in all domains for the 1999–2000 and 2004–2005 surveys, in English and Italian. These physical activity measures are not externally validated, but have been used in several national surveys and in different epidemiological studies (d’Errico et al. 2019; Franzoi et al. 2021; Gandini et al. 2019; Piccinelli et al. 2018). For OPA, in both surveys, employed participants were asked: ‘Is your work mostly characterised by physical activity?’. There were three response categories: ‘light activity, most of the time is spent sitting’, ‘moderate activity, most of the time I am on my feet’ or ‘heavy’ activity. Heavy activity was in the 1999–2000 survey defined as ‘enough to make me sweat for around half the time at work’. In the 2004–2005 survey, the corresponding response was ‘heavy activity, enough to make me sweat’. Domestic physical activity (DPA) was reported in four groups: none, light, moderate, or ‘heavy, enough to break a sweat’*.* For OPA and DPA, the questions were specific to these surveys. For LTPA, participants were asked separately about leisure-time activities corresponding to light activity, moderate activity, or intense activity. The wording differed between the two surveys: in the 1999–2000 survey, participants were asked to consider any activities in the past 12 months, and only responded the questions about ‘moderate’ or ‘light’ activities if they reported no leisure-time activities considered ‘intense’. In 2004–2005, participants were asked about activities performed at least once per week, and could report light, moderate, and intense activities independently. An overall LTPA classification was assigned based on the highest level of activity reported. Differences in the activities which counted towards leisure-time physical activity between the 1999–2000 and 2004–2005 questionnaires led to a different distribution of physical activity between cohorts. This was especially for the proportion of individuals reporting no leisure-time activity of any type (Table 1). Derivation of the categories is provided in appendix A. Full questionnaires are available from ISTAT (https://www.istat.it/it/archivio/5471).

**Table 1:** Descriptive characteristics of analytic sample

Survey	1999–2000 (*N* = 19,104)		2004–2005 (*N* = 21,116)		*P* for difference
	Median	(Range)	Median	(Range)	*p* (Wilcoxon-rank sum test)
Age	47	(40–55)	47	(40–55)	0.686
Physical Component Summary	55.19	(15.27–67.24)	55.26	(15.50–67.66)	< 0.001
Mental Component Summary	52.79	(9.51–70.55)	52.73	(7.54–69.13)	< 0.001
Chronic morbidity index	0.00	(0.00–58.89)	0.00	(0.00–45.98)	< 0.001

	%	%	*p* (*χ*^2^)
Gender			
Men	63.77	60.54	< 0.001
Women	36.23	39.46	
Family type			
Single	76.85	70.87	< 0.001
Couple with children	8.33	10.95	
Couple without children	8.16	9.80	
Single parents	6.66	8.37	
Area			
North	42.68	45.38	< 0.001
Central	19.06	19.21	
South and Islands	38.25	35.41	
OPA			
Light	35.56	35.43	0.192
Moderate	42.79	43.55	
Heavy	21.65	21.02	
DPA			
None	39.69	25.29	< 0.001
Light	14.01	21.43	
Moderate	35.37	45.62	
Heavy	10.93	7.66	
LTPA			
None	29.60	50.12	< 0.001
Light	32.14	22.76	
Moderate	27.98	18.82	
Intense	10.28	8.30	
Educational qualifications			
Post-diploma or degree	12.45	14.20	< 0.001
High school diploma	28.36	31.90	
Middle school diploma	39.79	42.78	
Primary school diploma or less	19.40	11.12	
Smoking status			
Never	33.19	29.31	< 0.001
Current	26.12	27.10	
Former smoker	40.69	43.59	
BMI			
< 18.5	0.97	1.38	< 0.001
18.5–24.9	48.45	50.92	
25.0–29.9	40.12	37.43	
≥ 30.0	10.46	10.27	
Diabetes			
No	97.83	97.68	0.300
Yes	2.17	2.32	

*OPA* occupational physical activity. *DPA* domestic physical activity. *LTPA* leisure-time physical activity. *BMI* body mass index

### Covariates

Analyses were gender-stratified, given previous evidence of differential associations for men and women. Age was included in years. Family type was defined as single, coupled with children, coupled without children, or single parents. To account for geographic area, we adjusted for major areas (Northern Italy, Central Italy, and the South and Islands) and clustered all analyses by region using STATA’s cluster() option. We included Physical Component Summary (PCS) and Mental Component Summary (MCS) index from the SF-12 questionnaire, both treated as continuous. A Chronic Morbidity Index (CMI) was constructed based on self-reported physician diagnoses of 22 chronic conditions, with details described elsewhere (d’Errico et al. 2019). This index was highly skewed, so it was categorized into five groups (participants with a CMI of 0, and quartiles of non-zero values). Smoking status was defined as never, ex-smoker or current smoker. Body mass index (BMI) was calculated from reported height and weight and categorised using WHO classifications. These were: underweight (< 18.5 kg/m^2^), recommended weight (the reference group, 18.5–24.9 kg/m^2^), overweight (25.0–29.9 kg/m^2^), and obese (30.0 kg/m^2^ or above). As a measure of socioeconomic position, we included a participant’s highest educational qualification (Post-diploma or degree/High school diploma/Middle school diploma/Primary school diploma or less).

### Statistical analyses

Missing data on exposures and covariates were imputed by ISTAT with automatic imputation methods. Details of the imputation process are provided at https://www.istat.it/en/methods-and-tools/methods-and-it-tools/process/processing-tools/concordjava. The proportion of imputed data points for the entire surveys was 2.5% in 1999–2000 and 2.8% in 2004–2005. All models were run separately for participants of the 1999–2000 and 2004–2005 surveys, due to differences in the phrasing of key questions or response categories between the surveys. Coefficients were then combined by random-effects meta-analysis using STATA’s meta package. Analyses were conducted using STATAv16.

Cox models were used to compare mortality between groups of OPA, LTPA, and DPA. Follow-up was defined as the period between baseline interview and date of event or censoring. Because risk of mortality and CHD increase considerably with age, in all models we included 3-year age group using STATA’s strata() option, thus allowing the baseline hazard function to differ between age groups. For DPA, different reference categories were used for men and women (‘none’ and ‘heavy’, respectively), due to very different distributions of DPA between genders (Table S1–S2). A base model adjusted for demographics (age in years, family type, and macroeconomic region). A second model added health (PCS, MCS, and the chronic morbidity index) health-related behaviours (smoking status and BMI categories), and highest educational qualification. A final model considered all types of physical activity together. In separate models, we included interaction terms between different domains of physical activity. We also stratified analyses by educational level, examining separately associations among participants with lower education (middle school diploma or less) and higher education (high school diploma or above). Schoenfeld tests were used to examine the proportional hazards assumption. To examine whether associations with all-cause mortality and CHD events changed during follow-up, additional models considered separately events in the first 5 years of follow-up, and events 5–14 years post-baseline (5–9 years post-baseline for the 2004–2005 cohort). In further sensitivity anal + yses, we excluded people with chronic disease at baseline (anyone with a non-zero value for the chronic morbidity index, *N* = 11,181 men, *N* = 8064 women), and participants who had changed jobs in the 5 years before baseline (*N* = 1723 men, *N* = 1746 women).

## Results

The 1999–2000 and 2004–2005 sample populations differed with respect to key variables. This included LTPA, where differences in the phrasing of the questions affected the distribution (Table 1). Smaller differences were seen for educational level, which was on average higher (*p* < 0.001) in the later survey. Participants of the 2004–2005 survey were less likely to smoke, reported lower BMI (*p* < 0.001) and less domestic physical activity (all *p* < 0.001). They did not differ substantially for OPA (*p* = 0.19). Descriptive characteristics of the sample by categories of OPA, DPA and LTPA are reported in Supplementary Tables S1 and S2. The number of deaths and CHD events in each group are shown in Supplementary Table S3.

### Association of occupational, domestic and leisure-time physical activity with all-cause mortality

**Fig. 1:**
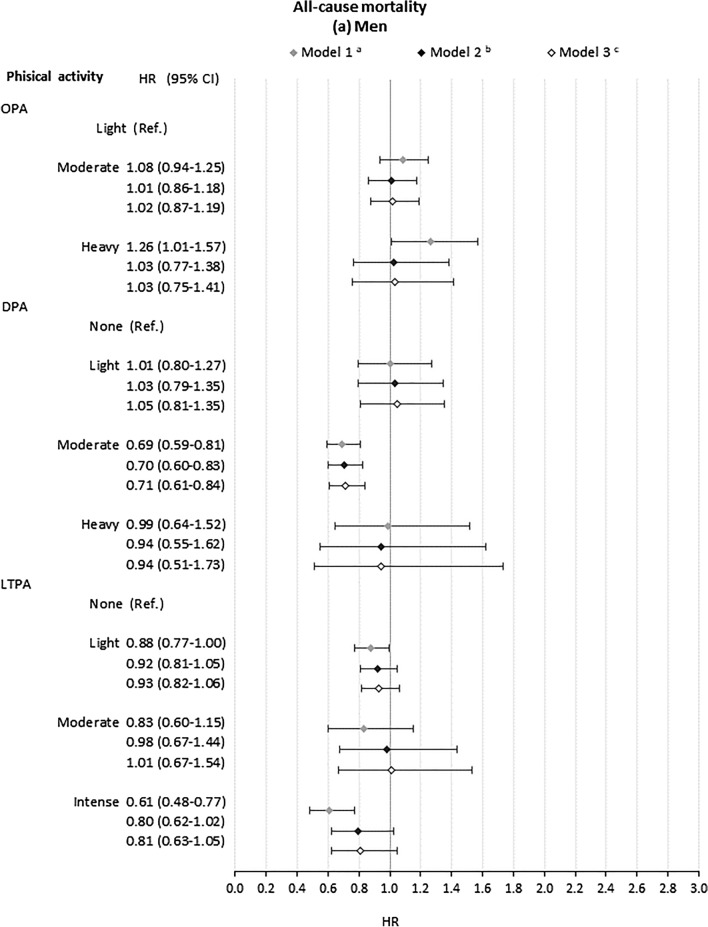

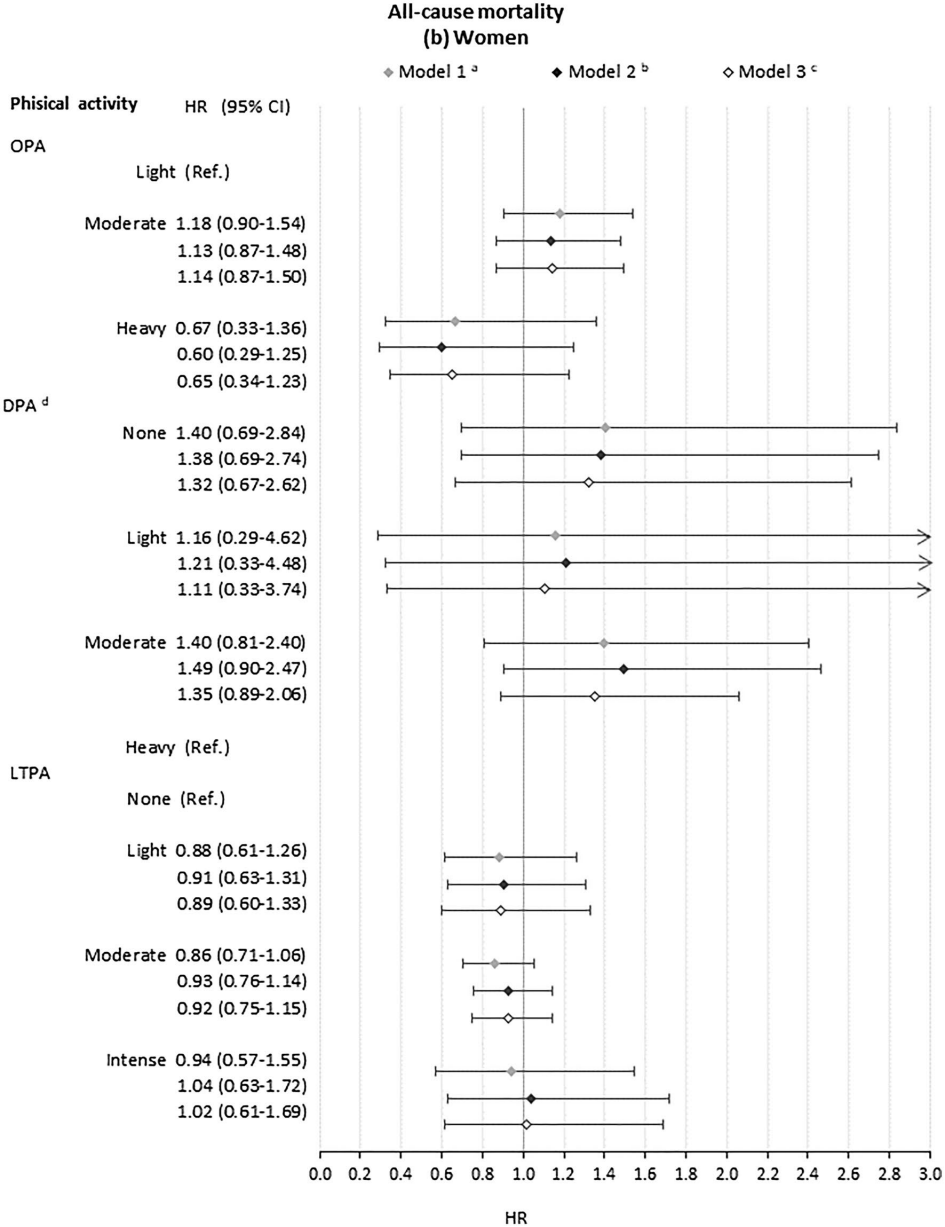
Association of domains of physical activity with all-cause mortality for **a** men and **b** women. *OPA* Occupational physical activity. *DPA* Domestic physical activity. *LTPA* leisure-time physical activity. ^a^Model 1: model including age, family type and macroeconomic region and each domain of physical activity separately. ^b^Model 2: Model 1 plus general health status(SF-12 Physical Component Summary, SF-12 Mental Component Summary, Chronic morbidity index categories), health behaviours (smoking status and BMI categories) and educational qualifications. ^c^Model 3: Model 2 plus adjustment for the other domains of physical activity. ^d^For women, high DPA was set as the baseline category due to small cell counts in the low activity group

In models adjusting only for age and geographic region (Fig. 1), heavy OPA compared to light OPA was associated with an increased risk of mortality among men (HR 1.26, CI 1.01, 1.57) but not women (HR 0.67, CI 0.33, 1.36). The increased risk for men disappeared with adjustment for health, health-related behaviours and education (HR 1.03, CI 0.77, 1.38). For neither gender was there strong evidence of increased risk associated with moderate OPA (Fig. 1). Moderate DPA, compared to no DPA, was associated with lower risk of mortality among men (HR 0.71, CI 0.61–0.84 with full adjustment). For women, there was no strong evidence that DPA associated with risk of mortality (Fig. 1). Intense LTPA, compared to no LTPA, was significantly associated with lower risk of mortality only for men in the crude model (HR 0.61 (CI 0.48, 0.77)). This attenuated with adjustment for confounders (HR 0.80 (CI 0.62–1.02)). Light and moderate LTPA, compared to no LTPA, were not associated with risk of mortality for men or women. Schoenfeld tests showed that the proportional hazards assumption was not met for all models (Supplementary Table S4). Interaction terms did not support interactive effects between different domains of physical activity (Supplementary Table S6).

### Association of occupational, domestic and leisure-time physical activity with CHD events

**Fig. 2:**
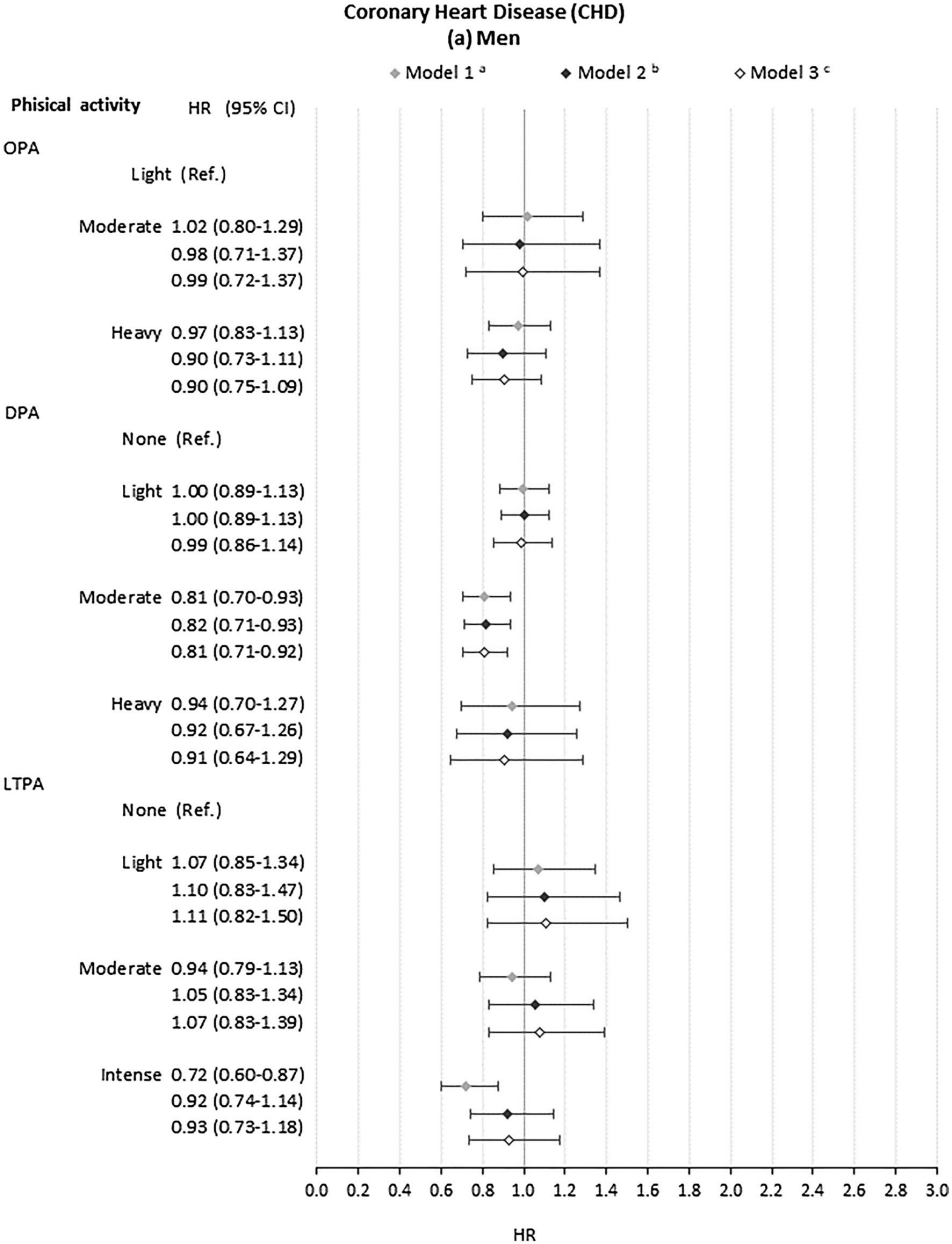

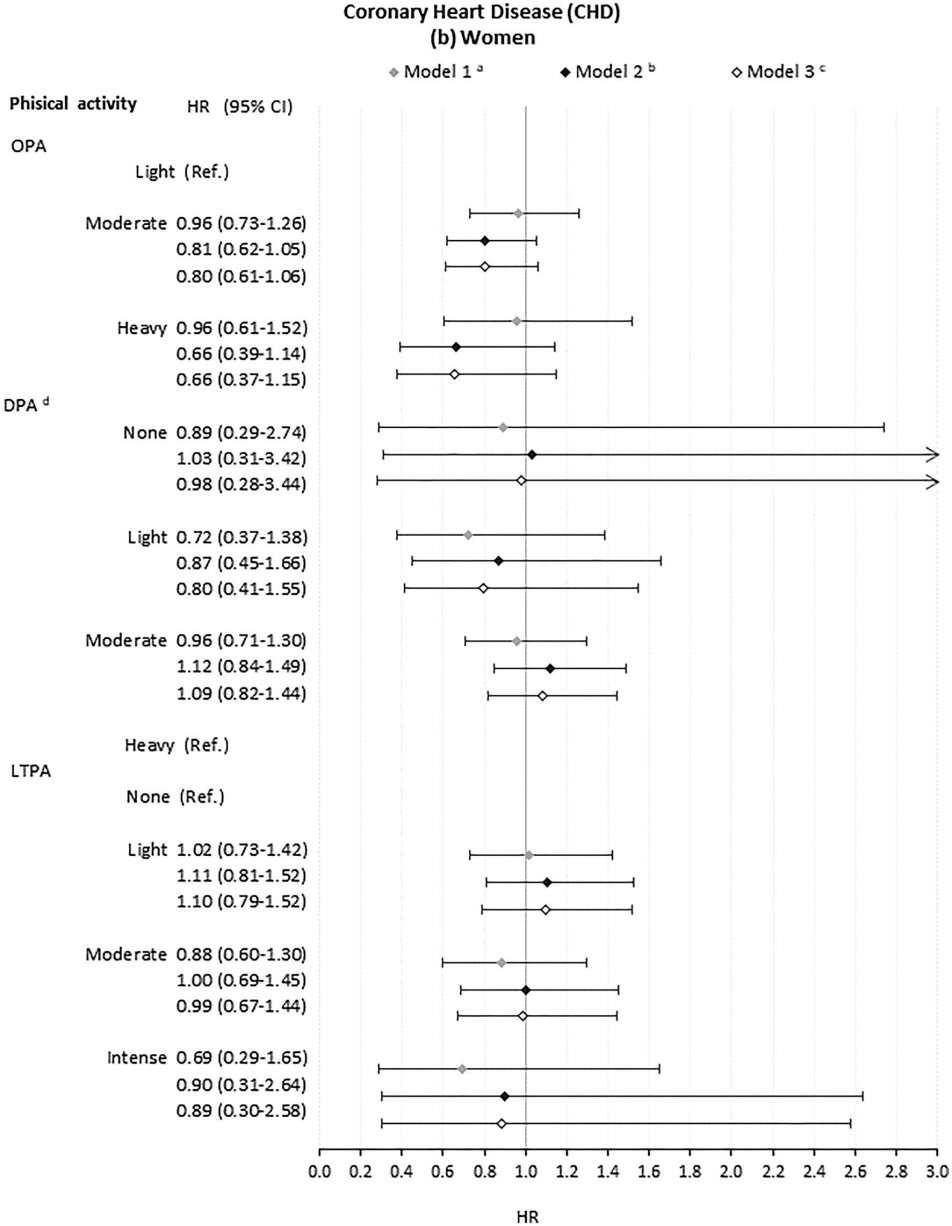
Association of domains of physical activity with Coronary Heart Disease (CHD) events for **a** men and **b** women. *OPA* occupational physical activity. *DPA* domestic physical activity. *LTPA* leisure-time physical activity. ^a^Model 1: model including age, family type and macroeconomic region and each domain of physical activity separately. ^b^Model 2: Model 1 plus general health status(SF-12 Physical Component Summary, SF-12 Mental Component Summary, Chronic morbidity index categories), health behaviours (smoking status and BMI categories) and educational qualifications. ^c^Model 3: Model 2 plus adjustment for the other domains of physical activity. ^d^For women, high DPA was set as the baseline category due to small cell counts in the low activity group

There was little evidence that OPA increased risk of CHD events for men or women at any level of adjustment (Fig. 2). Moderate DPA, compared to no DPA, was associated with lower risk of CHD events among men (HR 0.82, CI 0.72, 0.94) with full adjustment). For women, there was no strong evidence that DPA associated with risk of CHD events (Fig. 2b). Intense LTPA, compared to no LTPA, was significantly associated with decreased risk for men but not women in crude models (HR 0.72, CI 0.60, 0.87). This was attenuated with adjustment for health, health-related behaviours and education (HR 0.92, CI 0.74–1.14). Again, Schoenfeld tests showed that the proportional hazards assumption was not met for all models (Supplementary Table S5) and interaction terms did not support interactive effects between different domains of physical activity (Supplementary Table S7).

### Results of sensitivity analyses

When follow-up time was split into less than 5 years post-baseline and 5 or more years post-baseline, there was no strong evidence in either period of follow-up that OPA increased risk of mortality or CHD events for men or women (Supplementary Tables S8 and S9). Conclusions did not differ when the sample was also stratified by level of education (supplementary Tables S10 and S11). Excluding people with chronic disease at baseline (*N* = 11,181 men, N = 8064 women) reduced the number of events but did not alter conclusions for either outcome (Supplementary Tables S12 and S13). Excluding people who had changed jobs in the past 5 years (*N* = 1723 men, *N* = 1746 women) did not alter conclusions (Supplementary Tables S14 and S15).

## Discussion

This study considered whether OPA, DPA and LTPA are associated with all-cause mortality, and with CHD events, among middle-aged Italian workers. There was little evidence of increased risk with occupational physical activity for women, consistent with the majority of studies to date (Coenen et al. 2018; Dalene et al. 2021; Ervasti et al. 2019; Mikkola et al. 2019; Wanner et al. 2019). For men, the highest category of occupational physical activity was associated with increased risk of mortality, but not after adjustment for confounders. For neither gender was there strong evidence that OPA was associated with increased risk of CHD events. However, the modest size of our study compared to recent analyses (Dalene et al. 2021; Holtermann et al. 2021), and restriction to a younger age range in which mortality and CHD events are less common, meant that estimates were imprecise. Our results are therefore consistent with a range of effects of OPA on health, both beneficial and detrimental.

For men, our results contrast with pooled results from the 2018 meta-analysis, which reported an 18% increased risk of early mortality for the highest compared to the lowest OPA group. Besides the smaller sample size, the difference may reflect more rigorous adjustment in our study, since the meta-analysis included studies which adjusted only for age, gender and one other potential confounder. Our results also contrast with a recent analysis of 104,046 Danish men and women, in which an increased risk of mortality and cardiovascular events was robust to adjustment for extensive confounders (Holtermann et al. 2021), and a recent analysis of 437,378 Norwegian men and women, in which occupational physical activity was associated with greater longevity after full adjustment for men (Dalene et al. 2021). Again, the larger size of these studies meant they were better powered than ours to detect effects. However, other differences may have contributed. We restricted analysis to participants aged 40–55 at baseline to minimize influence of health-related selection, which would be expected to bias downwards associations of occupational physical activity with mortality and CHD events. Dalene et al. (2021) included workers up to age 65, and it is possible that the reported association of OPA with increased longevity partly reflects selection processes. Another difference concerns measurement of exposure. In our study and in both recent studies (Dalene et al. 2021; Holtermann et al. 2021), OPA was based on information reported by participants. Participant reports only partially capture true activity levels, and may bias towards the null associations of physical activity and health compared to accelerometer data (Guo et al. 2019). However, both Holtermann (Holtermann et al. 2021) and Dalene (Dalene et al. 2021) used a four-category measure of occupational physical activity based on the Saltin-Grimby Physical Activity Scale (Grimby et al. 2015). This differentiates between mostly sedentary work, work characterised by some walking, work characterised by walking and lifting, and heavy manual labour. In our study, the available measure of occupational physical activity had three categories and did not distinguish between walking and lifting. Because heavy activity was described as activity which made the participant sweat, responses may also have been influenced by higher ambient temperatures in a Mediterranean country. Additional misclassification of the exposure in our study may therefore have biased effects towards the null.

Separately, variation in socioeconomic patterning of health-related behaviours between countries may lead to different structures of residual confounding. Socioeconomic differences in dietary factors, including fruit and vegetable consumption, may be less pronounced in Mediterranean countries than in Northern Europe (Mertens et al. 2019; Prättälä et al. 2009). If associations of OPA with mortality and CHD events in part reflect residual confounding, less evidence of associations in Mediterranean countries might therefore be expected. Consistently, two other studies to examine this question in Italy found either little evidence that OPA harms health (Ferrario et al. 2018) or an association with increased longevity (Menotti et al. 2006), although both studies were small (*N* = 3574 and *N* = 1712). Given the risk of residual confounding by socioeconomic position and related psychosocial exposures, we would ideally have included income and job security as controls, but this information was not available. However, confounding by these factors would likely inflate associations of OPA with mortality and CHD events, so is unlikely to substantively explain our largely null results.

For men only, moderate DPA, compared to no DPA, was associated with lower risk of mortality and of CHD events. Since the risks associated with heavy DPA were estimated based on very few exposed cases, it was not possible to meaningfully assess if it was also protective or evaluate whether there was an inverse gradient in risk of mortality and CHD by DPA. Further studies will be required to fully understand the effects of DPA on health.

### Strengths and limitations

This study has several key strengths. Using data from a nationally representative survey population, we were able to adjust for important confounders, including educational qualifications, health-related behaviours, and health. However, the comparatively low number of mortality and CHD events in this age group substantially limited precision of estimates. Our results are therefore consistent with a range of effects, both detrimental and protective, of occupational physical activity on health. We restricted analysis to middle-aged workers to minimize influence of health-related selection, but retirement before age 55 may nevertheless have influenced results. We used data from nationally representative Italian surveys, but if structures of confounding vary between countries, results may not be fully generalizable to other country contexts. The proportional hazards assumption did not hold for all models although no strong evidence of time-varying HRs was found. Physical activity in all domains was based on reported information from a single time point, and it may not adequately capture activity levels throughout participants’ working lives. The item assessing OPA differed from other studies, and misclassification error in the exposure may have affected results. Although we adjusted for important confounding factors, residual confounding cannot be ruled out.

## Conclusion

In a sample of middle-aged Italian workers, we found limited evidence of harmful or beneficial health effects of occupational physical activity on mortality or CHD events for men or women. However, confidence intervals were wide, and results were consistent with a range of effects in both directions. More studies, with adequate adjustment and conducted in diverse country contexts, will be required to settle the question of the physical activity paradox.

## Supplementary Information

Below is the link to the electronic supplementary material.

Supplementary file1 (DO 256 KB)

Supplementary file2 (DOCX 154 KB)

## Data Availability

Data used for the analysis are subjected to the legal restrictions established by the European privacy law, as the data contain potentially identifying or sensitive patient information. Open access to data is not possible but collaborations in specific projects with other research groups or institutes are possible upon collaboration agreement approval from the Presidential Committee of the ISTAT. Further request of information on the project and on collaborations can be addressed to the principal investigator Gabriella Sebastiani (gabriella.sebastiani@istat.it).

## Code availability

STATA code used in the analysis is provided as a supplementary file.
