# Supplementary material for: Occupational physical activity, mortality and CHD events in the Italian Longitudinal Study
Source: Int Arch Occup Environ Health. 2021 Oct 11;95(3):607–19. doi: 10.1007/s00420-021-01765-0 (PMC8938372; doi:10.1007/s00420-021-01765-0)
Supplement: Supplementary file 2 — Supplementary file2 (DOCX 154 KB) [file 420_2021_1765_MOESM2_ESM.docx]

**Supplementary Materials**

**Occupational Physical Activity, Mortality and CHD events in the Italian Longitudinal Study**

Elena Strippoli^1^, Amanda Hughes^2^, Gabriella Sebastiani^3^, Paola Di Filippo^3^, Angelo d’Errico^1^

^1^ Epidemiology Department, Local Health Unit TO3, Piedmont Region, Grugliasco, Torino, Italy.

^2^ MRC Integrative Epidemiology Unit, University of Bristol, BS8 2BN, United Kingdom.

^3^ National Institute of Statistics (ISTAT), Rome.

Corresponding author details: Amanda Hughes, amanda.hughes@bristol.ac.uk

This manuscript has been submitted to the International Archives of Occupational and Environmental Health

**Index of contents**

**Table S1:** Distribution of population characteristics among men by domain of physical activity

**Table S2:** Distribution of population characteristics among women by domain of physical activity

**Table S3:** Number of mortality and Coronary Heart Disease (CHD) events by gender, survey, period of follow-up and domains of physical activity

**Table S4:** Tests of proportional hazards assumption for all-cause mortality

**Table S5:** Tests of proportional hazards assumption for Coronary Heart Disease (CHD) events

**Table S6:** Tests of interactions between domains of physical activity for all-cause mortality

**Table S7:** Tests of interactions between domains of physical activity for Coronary Heart Disease (CHD) events

**Table S8:** Gender-stratified association of domains of physical activity with all-cause mortality, stratified follow-up time

**Table S9:** Gender-stratified association of domains of physical activity with Coronary Heart Disease (CHD) events, stratified follow-up time

**Table 10:** Gender-stratified association of domains of physical activity with all-cause mortality

**Table 11:** Gender-stratified association of domains of physical activity with Coronary Heart Disease (CHD) events

**Table S12:** Gender-stratified association of domains of physical activity with all-cause mortality, excluding participants with any chronic illness at baseline

**Table S13:** Gender-stratified association of domains of physical activity with Coronary Heart Disease (CHD) events, excluding participants with any chronic illness at baseline

**Table S14:** Gender-stratified association of domains of physical activity with all-cause mortality, excluding participants who had changed jobs within the past 5 years

**Table S15**: Gender-stratified association of domains of physical activity with Coronary Heart Disease (CHD) events, excluding participants who had changed jobs within the past 5 years

**Appendix A:** Physical activity questions in the Italian National Health Interview Surveys

**Appendix B:** STROBE Statement—checklist of items that should be included in reports of observational studies

**Table S1: Distribution of population characteristics among men by domains of physical activity**

| **Men** | | **OPA** | | | **DPA** | | | | **LTPA** | | | |
| --- | --- | --- | --- | --- | --- | --- | --- | --- | --- | --- | --- | --- |
|  |  | Light | Moderate | Heavy | None | Light | Moderate | Heavy | None | Light | Moderate | Intense |
| **N subjects** | | 7,975 | 10,687 | 6,305 | 12,657 | 5,809 | 5,837 | 664 | 9,829 | 6,282 | 6,125 | 2,731 |
|  |  | **Median** | **Median** | **Median** | **Median** | **Median** | **Median** | **Median** | **Median** | **Median** | **Median** | **Median** |
|  |  | **(min-max)** | **(min-max)** | **(min-max)** | **(min-max)** | **(min-max)** | **(min-max)** | **(min-max)** | **(min-max)** | **(min-max)** | **(min-max)** | **(min-max)** |
| Age | | 47 | 47 | 47 | 47 | 46 | 46 | 47 | 47 | 47 | 47 | 45 |
|  |  | (40-55) | (40-55) | (40-55) | (40-55) | (40-55) | (40-55) | (40-55) | (40-55) | (40-55) | (40-55) | (40-55) |
| Physical functioning score | | 55.48 | 55.26 | 54.88 | 55.26 | 55.30 | 55.26 | 55.26 | 55.26 | 55.26 | 55.26 | 55.91 |
|  |  | (15.50-65.33) | (15.27-67.13) | (19.31-67.24) | (15.27-65.95) | (16.18-66.59) | (20.58-67.24) | (19.61-63.80) | (15.27-67.24) | (15.50-66.19) | (18.93-65.92) | (24.00-66.37) |
| Mental functioning score | | 52.79 | 52.90 | 52.82 | 52.85 | 52.82 | 52.82 | 52.73 | 52.79 | 52.82 | 52.99 | 53.37 |
|  |  | (8.77-69.60) | (9.51-70.55) | (9.51-68.70) | (9.51-69.60) | (8.77-70.55) | (9.51-68.70) | (12.58-67.11) | (9.51-70.55) | (8.77-68.70) | (9.51-69.13) | (12.66-67.37) |
| Chronic morbidity index | | 2.36 | 2.36 | 2.56 | 2.36 | 2.36 | 2.36 | 2.57 | 2.56 | 2.36 | 2.36 | 1.85 |
|  |  | (0.08-58.89) | (0.08-42.31) | (0.08-47.15) | (0.08-42.85) | (0.08-58.89) | (0.08-45.98) | (0.08-46.65) | (0.08-45.56) | (0.08-47.15) | (0.08-58.89) | (0.08-27.43) |
|  |  | **%** | **%** | **%** | **%** | **%** | **%** | **%** | **%** | **%** | **%** | **%** |
| Survey | 1999-2000 | 49.39 | 48.40 | 48.72 | 58.84 | 36.68 | 38.75 | 51.66 | 35.19 | 58.66 | 59.97 | 50.02 |
|  | 2004-2005 | 50.61 | 51.60 | 51.28 | 41.16 | 63.32 | 61.25 | 48.34 | 64.81 | 41.34 | 40.03 | 49.98 |
| Family type | Single | 76.65 | 77.44 | 79.76 | 83.22 | 74.73 | 69.56 | 72.89 | 79.27 | 78.80 | 75.76 | 74.59 |
|  | Couple with children | 10.71 | 9.74 | 8.79 | 5.64 | 12.39 | 16.19 | 10.54 | 8.85 | 9.28 | 10.84 | 12.16 |
|  | Couple no children | 8.73 | 8.28 | 7.41 | 7.45 | 8.69 | 9.01 | 11.14 | 7.65 | 7.88 | 8.73 | 9.74 |
|  | Single parents | 3.91 | 4.54 | 4.04 | 3.69 | 4.18 | 5.24 | 5.42 | 4.23 | 4.04 | 4.67 | 3.52 |
| Area | North | 42.19 | 43.31 | 40.79 | 36.54 | 44.36 | 51.36 | 55.12 | 35.89 | 40.74 | 52.02 | 47.35 |
|  | Central | 19.77 | 17.77 | 16.91 | 17.49 | 19.92 | 17.97 | 18.37 | 17.29 | 18.55 | 18.33 | 20.32 |
|  | South and Islands | 38.03 | 38.92 | 42.30 | 45.97 | 35.72 | 30.67 | 26.51 | 46.82 | 40.72 | 29.65 | 32.33 |
| Educational qualifications | Post-diploma or degree | 23.77 | 8.64 | 1.97 | 10.52 | 14.18 | 12.08 | 12.50 | 7.80 | 11.03 | 15.33 | 19.92 |
|  | High school diploma | 44.94 | 27.01 | 11.75 | 25.95 | 31.74 | 32.16 | 31.02 | 24.00 | 28.14 | 33.67 | 37.46 |
|  | Middle school diploma | 26.36 | 50.13 | 56.05 | 45.18 | 42.35 | 43.64 | 40.36 | 48.56 | 44.33 | 39.38 | 37.46 |
|  | Primary school diploma or less | 4.93 | 14.22 | 30.23 | 18.35 | 11.72 | 12.13 | 16.11 | 19.64 | 16.49 | 11.62 | 5.16 |
| Occupational social class | 1 | 35.66 | 18.39 | 10.17 | 21.85 | 23.58 | 19.68 | 25.00 | 17.55 | 20.47 | 25.73 | 31.60 |
|  | 2 | 42.37 | 20.73 | 5.00 | 19.99 | 26.89 | 28.23 | 25.45 | 18.96 | 24.53 | 28.00 | 28.89 |
|  | 3 | 7.22 | 19.09 | 28.52 | 21.55 | 14.36 | 13.00 | 14.16 | 21.07 | 17.11 | 14.42 | 14.10 |
|  | 4 | 13.67 | 40.73 | 54.86 | 35.62 | 33.79 | 37.71 | 34.49 | 40.77 | 37.06 | 31.04 | 24.39 |
|  | missing | 1.08 | 1.07 | 1.46 | 1.00 | 1.38 | 1.37 | 0.90 | 1.65 | 0.83 | 0.82 | 1.03 |
| Smoking status | Never | 32.41 | 33.35 | 38.64 | 35.81 | 33.67 | 32.09 | 33.58 | 39.45 | 35.94 | 29.18 | 24.24 |
|  | Current | 31.49 | 30.82 | 29.34 | 29.57 | 31.66 | 31.61 | 34.34 | 27.49 | 30.75 | 34.14 | 34.05 |
|  | Ex | 36.10 | 35.83 | 32.02 | 34.61 | 34.67 | 36.30 | 32.08 | 33.06 | 33.30 | 36.69 | 41.71 |
| BMI | <18.5 | 0.30 | 0.43 | 0.40 | 0.22 | 0.55 | 0.48 | 1.05 | 0.40 | 0.40 | 0.31 | 0.44 |
|  | 18.5-24.9 | 45.12 | 42.29 | 39.18 | 39.87 | 44.55 | 45.81 | 42.17 | 37.89 | 40.51 | 45.63 | 55.80 |
|  | 25.0-29.9 | 44.89 | 46.22 | 47.71 | 48.10 | 44.69 | 43.43 | 46.39 | 47.84 | 47.45 | 45.42 | 38.89 |
|  | ≥30.0 | 9.69 | 11.06 | 12.72 | 11.81 | 10.21 | 10.28 | 10.39 | 13.88 | 11.64 | 8.64 | 4.87 |
| Diabetes | No | 97.35 | 97.37 | 96.99 | 97.19 | 97.16 | 97.52 | 97.59 | 96.81 | 96.69 | 97.80 | 99.08 |
|  | Yes | 2.65 | 2.63 | 3.01 | 2.81 | 2.84 | 2.48 | 2.41 | 3.19 | 3.31 | 2.20 | 0.92 |

OPA: Occupational physical activity. DPA: Domestic physical activity. LTPA: leisure-time physical activity. BMI: body mass index

**Table S2: Distribution of population characteristics among women by domains of physical activity**

| **Women** | | **OPA** | | | **DPA** | | | | **LTPA** | | | |
| --- | --- | --- | --- | --- | --- | --- | --- | --- | --- | --- | --- | --- |
|  |  | None | Light | Heavy | None | Light | Moderate | Heavy | None | Light | Moderate | Intense |
| **N subjects** | | 6,300 | 6,684 | 2,269 | 265 | 1,393 | 10,554 | 3,041 | 6,408 | 4,665 | 3,194 | 986 |
|  |  | **Median** | **Median** | **Median** | **Median** | **Median** | **Median** | **Median** | **Median** | **Median** | **Median** | **Median** |
|  |  | **(min-max)** | **(min-max)** | **(min-max)** | **(min-max)** | **(min-max)** | **(min-max)** | **(min-max)** | **(min-max)** | **(min-max)** | **(min-max)** | **(min-max)** |
| Age | | 46 | 46 | 47 | 46 | 46 | 46 | 47 | 47 | 47 | 46 | 44 |
|  |  | (40-55) | (40-55) | (40-55) | (40-55) | (40-55) | (40-55) | (40-55) | (40-55) | (40-55) | (40-55) | (40-55) |
| Physical functioning score | | 55.26 | 54.83 | 53.12 | 55.30 | 55.26 | 54.84 | 54.21 | 54.72 | 54.64 | 55.19 | 55.36 |
|  |  | (17.74-67.66) | (19.29-65.18) | (17.84-67.26) | (21.33-63.83) | (21.65-65.15) | (17.85-67.34) | (17.74-67.66) | (17.85-67.66) | (17.84-66.19) | (17.74-66.10) | (20.39-67.19) |
| Mental functioning score | | 52.25 | 52.73 | 51.06 | 52.00 | 52.73 | 52.73 | 50.85 | 52.00 | 52.73 | 52.73 | 52.73 |
|  |  | (7.54-68.89) | (10.16-69.33) | (10.94-67.37) | (20.63-65.29) | (12.51-68.89) | (7.54-69.33) | (8.77-68.05) | (7.54-67.89) | (8.77-68.89) | (11.79-69.33) | (12.61-67.37) |
| Chronic morbidity index | | 2.83 | 2.93 | 3.60 | 3.60 | 3.01 | 2.79 | 3.53 | 3.47 | 2.91 | 2.75 | 2.36 |
|  |  | (0.08-44.93) | (0.08-52.62) | (0.08-40.88) | (0.08-44.54) | (0.08-37.31) | (0.08-44.93) | (0.08-52.62) | (0.08-44.93) | (0.08-52.62) | (0.08-40.88) | (0.08-39.48) |
|  |  | **%** | **%** | **%** | **%** | **%** | **%** | **%** | **%** | **%** | **%** | **%** |
| Survey | 1999-2000 | 45.32 | 44.91 | 46.89 | 50.94 | 39.12 | 42.60 | 57.38 | 34.25 | 52.63 | 52.38 | 60.65 |
|  | 2004-2005 | 54.68 | 55.09 | 53.11 | 49.06 | 60.88 | 57.40 | 42.62 | 65.75 | 47.37 | 47.62 | 39.35 |
| Family type | Single | 67.75 | 66.62 | 66.42 | 48.68 | 52.84 | 66.84 | 75.93 | 69.26 | 66.82 | 64.93 | 60.75 |
|  | Couple with children | 9.81 | 9.78 | 8.07 | 19.62 | 18.31 | 9.44 | 5.00 | 7.79 | 9.95 | 11.43 | 12.88 |
|  | Couple no children | 9.32 | 10.86 | 11.81 | 7.17 | 11.70 | 10.88 | 8.25 | 9.78 | 10.16 | 11.02 | 12.98 |
|  | Single parents | 13.13 | 12.73 | 13.71 | 24.53 | 17.16 | 12.85 | 10.82 | 13.17 | 13.08 | 12.62 | 13.39 |
| Area | North | 46.02 | 48.29 | 45.97 | 49.06 | 50.47 | 47.36 | 44.03 | 40.26 | 46.07 | 60.24 | 52.43 |
|  | Central | 21.32 | 20.33 | 20.01 | 19.25 | 21.90 | 20.51 | 20.88 | 20.66 | 22.02 | 19.04 | 19.98 |
|  | South and Islands | 32.67 | 31.37 | 34.02 | 31.70 | 27.64 | 32.13 | 35.09 | 39.08 | 31.92 | 20.73 | 27.59 |
| Educational qualifications | Post-diploma or degree | 26.30 | 10.41 | 3.53 | 23.02 | 25.05 | 16.09 | 10.69 | 12.55 | 16.38 | 18.75 | 26.98 |
|  | High school diploma | 45.48 | 26.45 | 13.62 | 33.96 | 37.98 | 32.02 | 31.04 | 29.90 | 32.65 | 35.60 | 37.12 |
|  | Middle school diploma | 24.02 | 45.17 | 48.92 | 32.45 | 28.00 | 37.68 | 39.10 | 39.93 | 35.76 | 34.78 | 30.83 |
|  | Primary school diploma or less | 4.21 | 17.97 | 33.94 | 10.57 | 8.97 | 14.21 | 19.17 | 17.62 | 15.22 | 10.86 | 5.07 |
| Occupational social class | 1 | 19.62 | 11.16 | 5.33 | 24.53 | 23.26 | 13.49 | 9.54 | 11.10 | 14.13 | 16.28 | 21.60 |
|  | 2 | 65.08 | 29.92 | 12.56 | 37.74 | 44.94 | 42.22 | 39.56 | 38.23 | 41.99 | 46.09 | 51.12 |
|  | 3 | 5.35 | 20.05 | 22.43 | 14.34 | 11.20 | 14.36 | 15.65 | 16.20 | 14.08 | 12.02 | 10.85 |
|  | 4 | 8.06 | 36.07 | 56.02 | 18.49 | 18.45 | 27.35 | 32.82 | 31.43 | 27.44 | 23.61 | 14.40 |
|  | missing | 1.89 | 2.80 | 3.66 | 4.91 | 2.15 | 2.58 | 2.43 | 3.04 | 2.36 | 2.00 | 2.03 |
| Smoking status | Never | 25.29 | 25.94 | 27.24 | 30.57 | 29.36 | 25.01 | 26.80 | 26.73 | 25.64 | 24.67 | 25.15 |
|  | Current | 22.29 | 19.00 | 16.88 | 17.36 | 20.75 | 20.04 | 19.96 | 17.01 | 19.87 | 24.17 | 27.18 |
|  | Ex | 52.43 | 55.06 | 55.88 | 52.08 | 49.89 | 54.95 | 53.24 | 56.26 | 54.49 | 51.16 | 47.67 |
| BMI | <18.5 | 2.83 | 2.38 | 1.98 | 5.66 | 3.16 | 2.36 | 2.43 | 2.36 | 2.25 | 2.85 | 3.55 |
|  | 18.5-24.9 | 67.78 | 60.02 | 50.15 | 65.28 | 67.19 | 62.63 | 55.94 | 56.16 | 61.91 | 68.85 | 74.44 |
|  | 25.0-29.9 | 22.33 | 28.19 | 33.05 | 21.89 | 21.18 | 26.48 | 29.37 | 29.63 | 26.67 | 22.23 | 19.07 |
|  | ≥30.0 | 7.06 | 9.41 | 14.81 | 7.17 | 8.47 | 8.53 | 12.27 | 11.84 | 9.17 | 6.07 | 2.94 |
| Diabetes | No | 98.92 | 98.47 | 97.71 | 98.11 | 98.28 | 98.71 | 98.13 | 98.39 | 98.37 | 98.84 | 99.39 |
|  | Yes | 1.08 | 1.53 | 2.29 | 1.89 | 1.72 | 1.29 | 1.87 | 1.61 | 1.63 | 1.16 | 0.61 |

OPA: Occupational physical activity. DPA: Domestic physical activity. LTPA: leisure-time physical activity. BMI: body mass index

**Table S3: Length of follow-up, number of mortality and Coronary Heart Disease (CHD) events by gender, survey, period of follow-up and domains of physical activity**

|  | **All-cause mortality** | | | | **CHD** | | | |  |
| --- | --- | --- | --- | --- | --- | --- | --- | --- | --- |
|  | **1999-2000 survey** | | **2005 survey** | | **1999-2000 survey** | | **2005 survey** | |  |
| **Total number of events** |  | |  | |  | |  | |  |
| Men | 804 | | 312 | | 909 | | 533 | |  |
| Women | 204 | | 110 | | 113 | | 94 | |  |
| **Total length of follow-up** |  | |  | |  | |  | |  |
| Men | 177,245 | | 122,241 | | 172,081 | | 120,038 | |  |
| Women | 102,026 | | 80,122.50 | | 101,399 | | 79,797 | |  |
| **Average length (standard deviation) of follow-up** |  | |  | |  | |  | |  |
| Men | 14.55 (1.74) | | 9.56 (0.79) | | 14.12 (2.54) | | 9.39 (1.27) | |  |
| Women | 14.74 (1.24) | | 9.62 (0.54) | | 14.65 (1.51) | | 9.58 (0.71) | |  |
| *Follow-up period* | <5 years | 5≥ years | <5 years | 5≥ years | <5 years | 5≥ years | <5 years | 5≥ years |  |
| **OPA** | | | | | | | | | |
| **Men** |  |  |  |  |  |  |  |  |  |
| Light OPA | 34 | 217 | 32 | 58 | 70 | 212 | 100 | 84 |  |
| Moderate OPA | 58 | 277 | 50 | 79 | 108 | 303 | 98 | 116 |  |
| Heavy OPA | 45 | 173 | 32 | 61 | 58 | 158 | 52 | 83 |  |
| **Women** |  |  |  |  |  |  |  |  |  |
| Light OPA | 18 | 54 | 14 | 35 | 7 | 39 | 12 | 26 |  |
| Moderate OPA | 18 | 87 | 17 | 36 | 10 | 36 | 19 | 26 |  |
| Heavy OPA | 9 | 18 | 0 | 8 | 2 | 19 | 2 | 9 |  |
| **DPA** | | | | | | | | | |
| **Men** |  |  |  |  |  |  |  |  |  |
| None DPA | 82 | 429 | 50 | 94 | 163 | 412 | 119 | 128 |  |
| Light DPA | 24 | 135 | 31 | 59 | 39 | 130 | 68 | 87 |  |
| Moderate DPA | 25 | 83 | 31 | 41 | 27 | 112 | 61 | 59 |  |
| Heavy DPA | 6 | 20 | 2 | 4 | 7 | 19 | 2 | 9 |  |
| **Women** |  |  |  |  |  |  |  |  |  |
| None DPA | 1 | 5 | 0 | 1 | 0 | 1 | 1 | 1 |  |
| Light DPA | 3 | 6 | 5 | 9 | 0 | 7 | 4 | 3 |  |
| Moderate DPA | 30 | 112 | 25 | 61 | 15 | 60 | 24 | 45 |  |
| Heavy DPA | 11 | 36 | 1 | 8 | 4 | 26 | 4 | 12 |  |
| **LTPA** | | | | | | | | | |
| **Men** |  |  |  |  |  |  |  |  |  |
| None LTPA | 49 | 229 | 66 | 101 | 74 | 211 | 127 | 139 |  |
| Light LTPA | 43 | 223 | 23 | 35 | 78 | 208 | 60 | 75 |  |
| Moderate LTPA | 37 | 167 | 16 | 48 | 73 | 193 | 44 | 51 |  |
| Intense LTPA | 8 | 48 | 9 | 14 | 11 | 61 | 19 | 18 |  |
| **Women** |  |  |  |  |  |  |  |  |  |
| None LTPA | 20 | 46 | 18 | 42 | 6 | 36 | 18 | 29 |  |
| Light LTPA | 16 | 62 | 5 | 18 | 7 | 36 | 10 | 18 |  |
| Moderate LTPA | 8 | 37 | 7 | 15 | 4 | 20 | 5 | 10 |  |
| Intense LTPA | 1 | 14 | 1 | 4 | 2 | 2 | 0 | 4 |  |

OPA: Occupational physical activity. DPA: Domestic physical activity. LTPA: leisure-time physical activity

**Table S4: Tests of proportional hazards assumption for all-cause mortality**

|  |  | **Model 1^a^** | |  | **Model 2^b^** | |  | **Model 3^c^** | |
| --- | --- | --- | --- | --- | --- | --- | --- | --- | --- |
| **Survey** |  | **1999-2000** | **2004-2005** |  | **1999-2000** | **2004-2005** |  | **1999-2000** | **2004-2005** |
|  |  | **rho (p-value)** | **rho (p-value)** |  | **rho (p-value)** | **rho (p-value)** |  | **rho (p-value)** | **rho (p-value)** |
| **OPA** | |  |  |  |  |  |  |  |  |
| **Men** | Light OPA | Ref. | Ref. |  | Ref. | Ref. |  | Ref. | Ref. |
|  | Moderate OPA | -0.09 (<0.01) | 0.05 (0.53) |  | -0.06 (<0.01) | -0.01 (0.90) |  | -0.06 (<0.01) | -0.02 (0.65) |
|  | Heavy OPA | -0.10 (<0.01) | -0.04 (0.46) |  | -0.05 (<0.01) | -0.06 (0.03) |  | -0.04 (<0.01) | -0.07 (<0.01) |
| **Women** | Light OPA | Ref. | Ref. |  | Ref. | Ref. |  | Ref. | Ref. |
|  | Moderate OPA | 0.02 (0.75) | 0.03 (0.71) |  | 0.03 (0.49) | 0.06 (0.41) |  | 0.01 (0.83) | 0.10 (0.09) |
|  | Heavy OPA | -0.08 (0.13) | 0.14 (0.11) |  | -0.02 (0.55) | 0.25 (<0.01) |  | -0.04 (0.25) | 0.22 (<0.01) |
| **DPA** | |  |  |  |  |  |  |  |  |
| **Men** | None DPA | Ref. | Ref. |  | Ref. | Ref. |  | Ref. | Ref. |
|  | Light DPA | 0.05 (0.08) | -0.01 (0.82) |  | 0.01 (0.69) | 0.05 (0.06) |  | 0.03 (0.16) | 0.02 (0.50) |
|  | Moderate DPA | -0.06 (0.05) | -0.01 (0.75) |  | -0.06 (0.03) | 0.08 (0.01) |  | -0.05 (0.05) | 0.05 (0.06) |
|  | Heavy DPA | -0.09 (<0.01) | 0.03 (0.53) |  | -0.08 (<0.01) | 0.09 (0.02) |  | -0.07 (<0.01) | 0.09 (0.03) |
| **Women** | None DPA | -0.10 (0.13) | 0.07 (0.43) |  | -0.12 (0.03) | 0.19 (<0.01) |  | -0.14 (<0.01) | 0.20 (<0.01) |
|  | Light DPA | -0.06 (0.40) | -0.09 (0.20) |  | -0.02 (0.70) | 0.03 (0.62) |  | 0.00 (0.99) | 0.05 (0.37) |
|  | Moderate DPA | 0.03 (0.63) | -0.07 (0.30) |  | 0.08 (0.12) | 0.04 (0.46) |  | 0.06 (0.26) | 0.06 (0.30) |
|  | Heavy DPA | Ref. | Ref. |  | Ref. | Ref. |  | Ref. | Ref. |
| **LTPA** | |  |  |  |  |  |  |  |  |
| **Men** | None LTPA | Ref. | Ref. |  | Ref. | Ref. |  | Ref. | Ref. |
|  | Light LTPA | 0.00 (0.93) | 0.07 (0.13) |  | 0.05 (0.02) | 0.09 (0.02) |  | 0.01 (0.60) | 0.08 (0.02) |
|  | Moderate LTPA | -0.04 (0.18) | 0.14 (<0.01) |  | 0.00 (0.87) | 0.17 (<0.01) |  | -0.02 (0.27) | 0.14 (<0.01) |
|  | Intense LTPA | 0.03 (0.25) | 0.08 (0.10) |  | 0.02 (0.31) | -0.01 (0.88) |  | -0.02 (0.39) | 0.00 (0.95) |
| **Women** | None LTPA | Ref. | Ref. |  | Ref. | Ref. |  | Ref. | Ref. |
|  | Light LTPA | 0.04 (0.47) | 0.07 (0.31) |  | 0.03 (0.54) | -0.02 (0.71) |  | 0.03 (0.37) | 0.02 (0.66) |
|  | Moderate LTPA | 0.18 (0.07) | 0.03 (0.64) |  | 0.17 (0.01) | -0.04 (0.45) |  | 0.10 (0.06) | -0.08 (0.09) |
|  | Intense LTPA | 0.09 (0.06) | 0.03 (0.73) |  | 0.12 (<0.01) | -0.12 (0.05) |  | 0.12 (<0.01) | -0.09 (0.12) |
|  |  |  |  |  |  |  |  |  |  |

OPA: Occupational physical activity. DPA: Domestic physical activity. LTPA: leisure-time physical activity

^a^ Model 1: model including age, family type and macroeconomic region and each domain of physical activity separately

^b^ Model 2: Model 1 plus general health status(SF-12 Physical Component Summary, SF-12 Mental Component Summary, Chronic morbidity index categories), health behaviours (smoking status and BMI categories) and educational qualification

^c^ Model 3: Model 2 plus adjustment for the other domains of physical activity

**Table S5: Tests of proportional hazards assumption for Coronary Heart Disease (CHD) events**

|  |  | **Model 1^a^** | |  | **Model 2^b^** | | |  | **Model 3^c^** | |  |
| --- | --- | --- | --- | --- | --- | --- | --- | --- | --- | --- | --- |
| **Survey** |  | **1999-2000** | **2004-2005** |  | **1999-2000** | **2004-2005** |  | | **1999-2000** | **2004-2005** | |
|  |  | **rho (p-value)** | **rho (p-value)** |  | **rho (p-value)** | **rho (p-value)** |  | | **rho (p-value)** | **rho (p-value)** | |
| **OPA** | |  |  |  |  |  |  | |  |  | |
| **Men** | Light OPA | Ref. | Ref. |  | Ref. | Ref. |  | | Ref. | Ref. | |
|  | Moderate OPA | -0.02 (0.63) | 0.09 (0.01) |  | 0.03 (0.17) | 0.11 (<0.01) |  | | 0.05 (0.01) | 0.08 (<0.01) | |
|  | Heavy OPA | 0.01 (0.58) | 0.11 (<0.01) |  | 0.01 (0.79) | 0.15 (<0.01) |  | | 0.01 (0.51) | 0.10 (<0.01) | |
| **Women** | Light OPA | Ref. | Ref. |  | Ref. | Ref. |  | | Ref. | Ref. | |
|  | Moderate OPA | 0.02 (0.83) | -0.06 (0.49) |  | -0.06 (0.38) | -0.06 (0.36) |  | | -0.01 (0.83) | -0.04 (0.46) | |
|  | Heavy OPA | 0.11 (0.14) | 0.10 (0.21) |  | 0.03 (0.60) | 0.14 (0.02) |  | | -0.02 (0.67) | 0.21 (<0.01) | |
| **DPA** | |  |  |  |  |  |  | |  |  | |
| **Men** | None DPA | Ref. | Ref. |  | Ref. | Ref. |  | | Ref. | Ref. | |
|  | Light DPA | 0.00 (0.99) | 0.05 (0.25) |  | 0.06 (<0.01) | -0.02 (0.55) |  | | 0.07 (<0.01) | -0.02 (0.55) | |
|  | Moderate DPA | 0.05 (0.08) | 0.01 (0.65) |  | 0.03 (0.12) | -0.04 (0.10) |  | | 0.04 (0.04) | -0.07 (<0.01) | |
|  | Heavy DPA | 0.05 (0.07) | -0.02 (0.58) |  | 0.09 (<0.01) | 0.06 (0.02) |  | | 0.09 (<0.01) | 0.08 (<0.01) | |
| **Women** | None DPA | 0.06 (0.45) | -0.32 (<0.01) |  | 0.15 (0.01) | -0.21 (<0.01) |  | | 0.17 (<0.01) | -0.21 (<0.01) | |
|  | Light DPA | 0.06 (0.43) | -0.18 (0.02) |  | 0.08 (0.18) | -0.13 (0.05) |  | | 0.03 (0.56) | -0.13 (0.04) | |
|  | Moderate DPA | -0.04 (0.70) | -0.10 (0.15) |  | -0.12 (0.04) | -0.05 (0.36) |  | | -0.11 (0.05) | -0.04 (0.37) | |
|  | Heavy DPA | Ref. | Ref. |  | Ref. | Ref. |  | | Ref. | Ref. | |
| **LTPA** | |  |  |  |  |  |  | |  |  | |
| **Men** | None LTPA | Ref. | Ref. |  | Ref. | Ref. |  | | Ref. | Ref. | |
|  | Light LTPA | -0.02 (0.48) | 0.01 (0.83) |  | -0.06 (0.01) | 0.02 (0.51) |  | | -0.07 (<0.01) | 0.01 (0.63) | |
|  | Moderate LTPA | -0.01 (0.61) | 0.02 (0.48) |  | -0.04 (0.03) | -0.04 (0.08) |  | | -0.04 (0.01) | -0.08 (<0.01) | |
|  | Intense LTPA | 0.01 (0.66) | 0.01 (0.83) |  | -0.02 (0.22) | -0.06 (0.03) |  | | -0.04 (0.04) | -0.09 (<0.01) | |
| **Women** | None LTPA | Ref. | Ref. |  | Ref. | Ref. |  | | Ref. | Ref. | |
|  | Light LTPA | -0.02 (0.80) | 0.05 (0.57) |  | 0.00 (0.95) | -0.04 (0.63) |  | | -0.01 (0.84) | -0.07 (0.24) | |
|  | Moderate LTPA | -0.09 (0.22) | 0.02 (0.86) |  | -0.03 (0.61) | 0.00 (0.97) |  | | -0.02 (0.72) | -0.15 (<0.01) | |
|  | Intense LTPA | -0.25 (<0.01) | 0.06 (0.55) |  | -0.19 (<0.01) | 0.03 (0.63) |  | | -0.16 (0.02) | -0.07 (0.22) | |
|  |  | Ref. | Ref. |  | Ref. | Ref. |  | | Ref. | Ref. | |

OPA: Occupational physical activity. DPA: Domestic physical activity. LTPA: leisure-time physical activity

^a^ Model 1: model including age, family type and macroeconomic region and each domain of physical activity separately

^b^ Model 2: Model 1 plus general health status(SF-12 Physical Component Summary, SF-12 Mental Component Summary, Chronic morbidity index categories), health behaviours (smoking status and BMI categories) and educational qualification

^c^ Model 3: Model 2 plus adjustment for the other domains of physical activity

**Table S6: Tests of interactions between domains of physical activity for all-cause mortality**

| **All cause mortality** |  |  |  |  |  |
| --- | --- | --- | --- | --- | --- |
|  | **Men** | |  | **Women** | |
| **Survey** | **1999-2000** | **2004-2005** |  | **1999-2000** | **2004-2005** |
|  | HR^a^ (95% CI) | HR^a^ (95% CI) |  | HR^a^ (95% CI) | HR^a^ (95% CI) |
| Light OPA | Ref | Ref |  | Ref | Ref |
| Moderate OPA | 0.88 (0.61-1.26) | 1.20 (0.91-1.57) |  | 1.09 (0.73-1.65) | 1.02 (0.63-1.65) |
| Heavy OPA | 0.82 (0.58-1.18) | 1.35 (0.95-1.92) |  | 0.77 (0.41-1.43) | 0.41 (0.18-0.91) |
|  |  |  |  |  |  |
| None-Light LTPA | Ref | Ref |  | Ref | Ref |
| Moderate-Intense LTPA | 0.75 (0.53-1.05) | 1.48 (0.90-2.43) |  | 0.66 (0.39-1.12) | 1.21 (0.71-2.05) |
|  |  |  |  |  |  |
| None-Light DPA | Ref | Ref |  | Ref | Ref |
| Moderate-Heavy DPA | 0.74 (0.62-0.89) | 0.73 (0.55-0.99) |  | 1.48 (0.95-2.30) | 0.94 (0.65-1.34) |
| Interaction terms |  |  |  |  |  |
| Moderate-Intense LTPA x Moderate OPA | 1.18 (0.76-1.83) | 0.73 (0.40-1.34) |  | 1.68 (0.98-2.89) | 1.04 (0.46-2.35) |
| Moderate-Intense LTPA x Heavy OPA | 1.22 (0.78-1.92) | 0.73 (0.37-1.42) |  | 1.20 (0.36-4.01) | 0.89 (0.11-7.39) |
|  |  |  |  |  |  |
| Wald test for the interaction terms (p-value) | 0.673 | 0.502 |  | 0.130 | 0.987 |
|  |  |  |  |  |  |
| Light OPA | Ref | Ref |  | Ref | Ref |
| Moderate OPA | 0.93 (0.71-1.21) | 1.07 (0.89-1.30) |  | 1.29 (0.86-1.93) | 1.03 (0.72-1.48) |
| Heavy OPA | 0.88 (0.66-1.16) | 1.22 (0.92-1.62) |  | 0.82 (0.45-1.50) | 0.40 (0.18-0.89) |
|  |  |  |  |  |  |
| None-Light DPA | Ref | Ref |  | Ref | Ref |
| Moderate-Heavy DPA | 0.73 (0.58-0.92) | 0.73 (0.50-1.08) |  | 1.45 (0.86-2.45) | 0.92 (0.59-1.43) |
| Interaction terms |  |  |  |  |  |
| None-Light LTPA | Ref | Ref |  | Ref | Ref |
| Moderate-Intense LTPA | 0.84 (0.68-1.04) | 1.20 (0.88-1.63) |  | 0.84 (0.29-2.46) | 1.15 (0.42-3.13) |
|  |  |  |  |  |  |
| Moderate-Intense LTPA x Moderate-Heavy DPA | 1.03 (0.66-1.61) | 1.01 (0.61-1.68) |  | 1.06 (0.37-3.08) | 1.07 (0.41-2.80) |
|  |  |  |  |  |  |
| Wald test for the interaction terms (p-value) | 0.902 | 0.956 |  | 0.911 | 0.888 |
|  |  |  |  |  |  |
| Light OPA | Ref | Ref |  | Ref | Ref |
| Moderate OPA | 0.92 (0.71-1.20) | 1.04 (0.78-1.39) |  | 1.93 (0.65-5.69) | 1.63 (0.46-5.74) |
| Heavy OPA | 0.81 (0.62-1.06) | 1.11 (0.79-1.56) |  | 0.92 (0.11-7.75) | 1.01 (0.09-11.67) |
|  |  |  |  |  |  |
| None-Light DPA | Ref | Ref |  | Ref | Ref |
| Moderate-Heavy DPA | 0.64 (0.47-0.88) | 0.61 (0.37-0.99) |  | 1.85 (0.94-3.64) | 1.24 (0.57-2.72) |
|  |  |  |  |  |  |
| None-Light LTPA | Ref | Ref |  | Ref | Ref |
| Moderate-Intense LTPA | 0.84 (0.71-1.00) | 1.20 (0.95-1.52) |  | 0.89 (0.67-1.19) | 1.22 (0.72-2.08) |
| Interaction terms |  |  |  |  |  |
| Moderate-Heavy DPA x Moderate OPA | 1.04 (0.72-1.50) | 1.17 (0.60-2.26) |  | 0.64 (0.22-1.91) | 0.59 (0.14-2.47) |
| Moderate-Heavy DPA x Heavy OPA | 1.60 (1.02-2.51) | 1.49 (0.85-2.60) |  | 0.87 (0.11-7.04) | 0.35 (0.03-4.74) |
|  |  |  |  |  |  |
| Wald test for the interaction terms (p-value) | 0.078 | 0.359 |  | 0.728 | 0.680 |
|  |  |  |  |  |  |

OPA: Occupational physical activity. DPA: Domestic physical activity. LTPA: leisure-time physical activity

^a^ Models including all domains of physical activity and interaction terms plus age, family type and macroeconomic region, general health status(SF-12 Physical Component Summary, SF-12 Mental Component Summary, Chronic morbidity index categories), health behaviours (smoking status and BMI categories) and educational qualification

**Table S7: Tests of interactions between domains of physical activity for Coronary Heart Disease (CHD) events**

| **CHD** |  |  |  |  |  |
| --- | --- | --- | --- | --- | --- |
|  | **Men** | |  | **Women** | |
| **Survey** | **1999-2000** | **2004-2005** |  | **1999-2000** | **2004-2005** |
|  | HR^a^ (95% CI) | HR^a^ (95% CI) |  | HR^a^ (95% CI) | HR^a^ (95% CI) |
| Light OPA | Ref | Ref |  | Ref | Ref |
| Moderate OPA | 1.27 (1.07-1.51) | 0.89 (0.68-1.15) |  | 0.78 (0.51-1.19) | 0.97 (0.58-1.63) |
| Heavy OPA | 0.97 (0.80-1.17) | 0.85 (0.63-1.15) |  | 0.68 (0.35-1.29) | 0.34 (0.14-0.84) |
|  |  |  |  |  |  |
| None-Light LTPA | Ref | Ref |  | Ref | Ref |
| Moderate-Intense LTPA | 1.06 (0.80-1.42) | 1.29 (0.95-1.74) |  | 0.76 (0.39-1.48) | 1.05 (0.48-2.28) |
|  |  |  |  |  |  |
| None-Light DPA | Ref | Ref |  | Ref | Ref |
| Moderate-Heavy DPA | 0.83 (0.70-0.98) | 0.81 (0.66-0.99) |  | 1.40 (0.64-3.04) | 1.26 (0.64-2.47) |
| Interaction terms |  |  |  |  |  |
| Moderate-Intense LTPA x Moderate OPA | 0.78 (0.57-1.07) | 0.78 (0.51-1.19) |  | 0.82 (0.33-2.04) | 0.67 (0.26-1.78) |
| Moderate-Intense LTPA x Heavy OPA | 1.07 (0.75-1.54) | 0.74 (0.43-1.28) |  | 2.10 (0.54-8.18) | 4.32 (1.05-17.75) |
|  |  |  |  |  |  |
| Wald test for the interaction terms (p-value) | 0.082 | 0.411 |  | 0.235 | 0.047 |
|  |  |  |  |  |  |
| Light OPA | Ref | Ref |  | Ref | Ref |
| Moderate OPA | 1.15 (1.01-1.31) | 0.83 (0.65-1.05) |  | 0.75 (0.53-1.05) | 0.91 (0.57-1.45) |
| Heavy OPA | 0.98 (0.80-1.20) | 0.80 (0.61-1.04) |  | 0.83 (0.45-1.52) | 0.47 (0.22-1.00) |
|  |  |  |  |  |  |
| None-Light DPA | Ref | Ref |  | Ref | Ref |
| Moderate-Heavy DPA | 0.84 (0.69-1.02) | 0.72 (0.54-0.97) |  | 1.47 (0.63-3.45) | 1.50 (0.74-3.05) |
| Interaction terms |  |  |  |  |  |
| No-Light LTPA | Ref | Ref |  | Ref | Ref |
| Moderate-Intense LTPA | 0.97 (0.83-1.13) | 0.99 (0.72-1.37) |  | 0.99 (0.11-8.63) | 1.98 (0.74-5.32) |
|  |  |  |  |  |  |
| Moderate-Intense LTPA x Moderate-Heavy DPA | 0.99 (0.77-1.26) | 1.42 (0.76-2.68) |  | 0.81 (0.08-8.70) | 0.50 (0.15-1.65) |
|  |  |  |  |  |  |
| Wald test for the interaction terms (p-value) | 0.909 | 0.275 |  | 0.865 | 0.255 |
|  |  |  |  |  |  |
| Light OPA | Ref | Ref |  | Ref | Ref |
| Moderate OPA | 1.14 (0.99-1.31) | 0.78 (0.58-1.05) |  | 0.71 (0.21-2.40) | 0.43 (0.10-1.78) |
| Heavy OPA | 0.95 (0.78-1.17) | 0.77 (0.57-1.05) |  | ^b^ | 0.69 (0.07-6.63) |
|  |  |  |  |  |  |
| None-Light DPA | Ref | Ref |  | Ref | Ref |
| Moderate-Heavy DPA | 0.79 (0.63-0.99) | 0.70 (0.50-1.00) |  | 1.20 (0.47-3.08) | 1.00 (0.41-2.45) |
|  |  |  |  |  |  |
| None-Light LTPA | Ref | Ref |  | Ref | Ref |
| Moderate-Intense LTPA | 0.96 (0.84-1.11) | 1.10 (0.87-1.40) |  | 0.81 (0.54-1.23) | 1.08 (0.62-1.88) |
| Interaction terms |  |  |  |  |  |
| Moderate-Heavy DPA x Moderate OPA | 1.05 (0.72-1.52) | 1.26 (0.74-2.13) |  | 1.06 (0.32-3.54) | 2.29 (0.53-9.90) |
| Moderate-Heavy DPA x Heavy OPA | 1.16 (0.80-1.69) | 1.16 (0.69-1.93) |  | ^b^ | 0.67 (0.06-7.13) |
|  |  |  |  |  |  |
| Wald test for the interaction terms (p-value) | 0.728 | 0.691 |  | 0.920 | 0.454 |
|  |  |  |  |  |  |

OPA: Occupational physical activity. DPA: Domestic physical activity. LTPA: leisure-time physical activity

^a^ Models including all domains of physical activity and interaction terms plus age, family type and macroeconomic region, general health status(SF-12 Physical Component Summary, SF-12 Mental Component Summary, Chronic morbidity index categories), health behaviours (smoking status and BMI categories) and educational qualification

^b^ Coefficients could not be calculated because in both surveys due to lack of events in this category

**Table S8: Gender-stratified association of domains of physical activity with all-cause mortality, stratified follow-up time**

|  | **Follow-up time: <5 years since baseline** | | |  | **Follow-up time: 5+ years since baseline** | | |
| --- | --- | --- | --- | --- | --- | --- | --- |
|  | **Model 1^a^** | **Model 2^b^** | **Model 3^c^** |  | **Model 1^a^** | **Model 2^b^** | **Model 3^c^** |
|  | **HR (95% CI)** | **HR (95% CI)** | **HR (95% CI)** |  | **HR (95% CI)** | **HR (95% CI)** | **HR (95% CI)** |
| **Men** |  |  |  |  |  |  |  |
| Light OPA | Ref | Ref | Ref |  | Ref | Ref | Ref |
| Moderate OPA | 1.26 (0.93-1.71) | 1.11 (0.79-1.56) | 1.11 (0.79-1.55) |  | 1.00 (0.82-1.23) | 0.93 (0.74-1.17) | 0.95 (0.75-1.19) |
| Heavy OPA | 1.52 (1.09-2.13) | 1.15 (0.78-1.70) | 1.14 (0.77-1.69) |  | 1.20 (0.88-1.64) | 1.02 (0.65-1.62) | 1.04 (0.63-1.69) |
| None DPA | Ref | Ref | Ref |  | Ref | Ref | Ref |
| Light DPA | 0.91 (0.67-1.23) | 0.95 (0.70-1.29) | 0.97 (0.72-1.30) |  | 1.06 (0.88-1.28) | 1.08 (0.86-1.35) | 1.09 (0.89-1.34) |
| Moderate DPA | 0.95 (0.67-1.33) | 0.98 (0.69-1.37) | 0.99 (0.70-1.39) |  | 0.62 (0.53-0.74) | 0.63 (0.53-0.75) | 0.64 (0.54-0.76) |
| Heavy DPA | 1.16 (0.44-3.04) | 1.07 (0.38-3.05) | 1.09 (0.38-3.09) |  | 0.95 (0.66-1.35) | 0.97 (0.70-1.33) | 0.97 (0.65-1.46) |
| None LTPA | Ref | Ref | Ref |  | Ref | Ref | Ref |
| Light LTPA | 0.81 (0.62-1.08) | 0.89 (0.67-1.17) | 0.88 (0.66-1.16) |  | 0.87 (0.77-1.00) | 0.91 (0.80-1.05) | 0.94 (0.81-1.08) |
| Moderate LTPA | 0.69 (0.51-0.93) | 0.86 (0.63-1.19) | 0.86 (0.62-1.18) |  | 0.93 (0.52-1.65) | 1.07 (0.59-1.93) | 1.11 (0.59-2.11) |
| Intense LTPA | 0.60 (0.37-1.00) | 0.88 (0.52-1.48) | 0.88 (0.52-1.50) |  | 0.62 (0.46-0.83) | 0.79 (0.58-1.07) | 0.80 (0.58-1.10) |
|  |  |  |  |  |  |  |  |
| **Women** |  |  |  |  |  |  |  |
| Light OPA | Ref | Ref | Ref |  | Ref | Ref | Ref |
| Moderate OPA | 1.04 (0.65-1.66) | 0.86 (0.55-1.33) | 0.87 (0.56-1.37) |  | 1.23 (0.82-1.84) | 1.25 (0.84-1.85) | 1.24 (0.84-1.82) |
| Heavy OPA | 1.31 (0.51-3.39) ^d^ | 0.80 (0.31-2.06) ^d^ | 0.70 (0.26-1,91) ^d^ |  | 0.72 (0.44-1.18) | 0.71 (0.41-1.25) | 0.75 (0.43-1.32) |
| None DPA | ^e^ | ^e^ | ^e^ |  | 1.46 (0.67-3.19) | 1.34 (0.62-2.92) | 1.30 (0.60-2.84) |
| Light DPA | 2.48 (0.31-19.95) | 2.95 (0.37-23.29) | 2.34 (0.38-14.45) |  | 0.93 (0.27-3.17) | 0.94 (0.30-2.92) | 0.90 (0.30-2.69) |
| Moderate DPA | 1.90 (0.41-8.80) | 2.14 (0.48-9.56) | 1.72 (0.53-5.60) |  | 1.23 (0.96-1.57) | 1.31 (1.03-1.68) | 1.24 (0.98-1.58) |
| Heavy DPA | Ref | Ref | Ref |  | Ref | Ref | Ref |
| None LTPA | Ref | Ref | Ref |  | Ref | Ref | Ref |
| Light LTPA | 0.59 (0.32-1.11) | 0.62 (0.33-1.19) | 0.62 (0.32-1.19) |  | 0.99 (0.67-1.46) | 1.02 (0.72-1.45) | 1.01 (0.70-1.45) |
| Moderate LTPA | 0.64 (0.32-1.25) | 0.68 (0.36-1.29) | 0.68 (0.35-1.30) |  | 0.98 (0.82-1.17) | 1.04 (0.88-1.24) | 1.03 (0.86-1.25) |
| Intense LTPA | 0.35 (0.08-1.60) | 0.40 (0.09-1.79) | 0.39 (0.09-1.73) |  | 1.17 (0.70-1.95) | 1.29 (0.76-2.20) | 1.28 (0.75-2.16) |
|  |  |  |  |  |  |  |  |

OPA: Occupational physical activity. DPA: Domestic physical activity. LTPA: leisure-time physical activity

^a^ Model 1: model including age, family type and macroeconomic region and each domain of physical activity separately

^b^ Model 2: Model 1 plus general health status(SF-12 Physical Component Summary, SF-12 Mental Component Summary, Chronic morbidity index categories), health behaviours (smoking status and BMI categories) and educational qualification

^c^ Model 3: Model 2 plus adjustment for the other domains of physical activity

^d^ Coefficients based only on the 1999-2000 survey. Coefficients could not be calculated in the 2004-2005 survey due to lack of events in this category

^e^ Coefficients could not be calculated because in both surveys due to lack of events in this category

**Table S9: Gender-stratified association of domains of physical activity with Coronary Heart Disease (CHD) events, stratified follow-up time**

|  | **Follow-up time: <5 years since baseline** | | |  | **Follow-up time: 5+ years since baseline** | | |
| --- | --- | --- | --- | --- | --- | --- | --- |
|  | **Model 1^a^** | **Model 2^b^** | **Model 3^c^** |  | **Model 1^a^** | **Model 2^b^** | **Model 3^c^** |
|  | **HR (95% CI)** | **HR (95% CI)** | **HR (95% CI)** |  | **HR (95% CI)** | **HR (95% CI)** | **HR (95% CI)** |
| **Men** |  |  |  |  |  |  |  |
| Light OPA | Ref | Ref | Ref |  | Ref | Ref | Ref |
| Moderate OPA | 0.94 (0.60-1.47) | 0.89 (0.50-1.56) | 0.89 (0.51-1.56) |  | 1.09 (0.94-1.25) | 1.09 (0.93-1.28) | 1.11 (0.94-1.30) |
| Heavy OPA | 0.84 (0.54-1.31) | 0.73 (0.39-1.36) | 0.73 (0.40-1.32) |  | 1.05 (0.82-1.35) | 1.00 (0.81-1.23) | 1.01 (0.83-1.24) |
| None DPA | Ref | Ref | Ref |  | Ref | Ref | Ref |
| Light DPA | 0.87 (0.73-1.03) | 0.87 (0.73-1.04) | 0.85 (0.71-1.02) |  | 1.09 (0.95-1.24) | 1.09 (0.96-1.25) | 1.08 (0.95-1.22) |
| Moderate DPA | 0.70 (0.47-1.05) | 0.71 (0.48-1.07) | 0.71 (0.49-1.04) |  | 0.87 (0.70-1.07) | 0.88 (0.73-1.05) | 0.86 (0.68-1.08) |
| Heavy DPA | 0.60 (0.20-1.81) | 0.56 (0.18-1.73) | 0.57 (0.19-1.73) |  | 1.16 (0.80-1.67) | 1.13 (0.78-1.63) | 1.11 (0.76-1.61) |
| None LTPA | Ref | Ref | Ref |  | Ref | Ref | Ref |
| Light LTPA | 1.06 (0.87-1.28) | 1.10 (0.91-1.33) | 1.11 (0.92-1.34) |  | 1.08 (0.78-1.49) | 1.12 (0.76-1.66) | 1.13 (0.74-1.74) |
| Moderate LTPA | 0.99 (0.74-1.31) | 1.14 (0.86-1.51) | 1.15 (0.87-1.53) |  | 0.92 (0.75-1.11) | 1.00 (0.78-1.28) | 1.03 (0.75-1.41) |
| Intense LTPA | 0.64 (0.34-1.20) | 0.84 (0.42-1.69) | 0.86 (0.43-1.69) |  | 0.76 (0.61-0.96) | 0.93 (0.75-1.14) | 0.92 (0.75-1.12) |
|  |  |  |  |  |  |  |  |
| **Women** |  |  |  |  |  |  |  |
| Light OPA | Ref | Ref | Ref |  | Ref | Ref | Ref |
| Moderate OPA | 1.43 (0.81-2.55) | 1.22 (0.63-2.35) | 1.23 (0.63-2.39) |  | 0.85 (0.62-1.17) | 0.68 (0.49-0.95) | 0.68 (0.48-0.95) |
| Heavy OPA | 0.63 (0.22-1.84) | 0.55 (0.15-1.97) | 0.57 (0.16-2.06) |  | 1.05 (0.65-1.69) | 0.73 (0.44-1.22) | 0.72 (0.43-1.22) |
| None DPA | 2.86 (0.25-32.36) ^d^ | 3.65 (0.28-46.81) ^d^ | 3.46 (0.28-43.16) ^d^ |  | 0.64 (0.16-2.54) | 0.76 (0.19-3.07) | 0.74 (0.19-2.92) |
| Light DPA | 1.72 (0.49-5.97) ^d^ | 2.31 (0.62-8.63) ^d^ | 1.94 (0.58-6.44) ^d^ |  | 0.69 (0.33-1.43) | 0.82 (0.37-1.81) | 0.76 (0.33-1.72) |
| Moderate DPA | 1.41 (0.72-2.77) | 1.81 (1.01-3.23) | 1.67 (0.92-3.02) |  | 0.87 (0.61-1.23) | 1.02 (0.73-1.42) | 0.99 (0.71-1.38) |
| Heavy DPA | Ref | Ref | Ref |  | Ref | Ref | Ref |
| None LTPA | Ref | Ref | Ref |  | Ref | Ref | Ref |
| Light LTPA | 1.04 (0.56-1.92) | 1.14 (0.62-2.11) | 1.14 (0.59-2.20) |  | 1.02 (0.69-1.50) | 1.08 (0.73-1.60) | 1.07 (0.72-1.60) |
| Moderate LTPA | 0.87 (0.36-2.09) | 0.94 (0.43-2.08) | 0.94 (0.42-2.07) |  | 0.91 (0.57-1.44) | 1.00 (0.62-1.61) | 0.98 (0.61-1.58) |
| Intense LTPA | 1.35 (0.23-7.72) ^e^ | 1.29 (0.24-6.85) ^e^ | 1.22 (0.22-6.80) ^e^ |  | 0.72 (0.11-4.53) | 0.93 (0.13-6.78) | 0.93 (0.13-6.60) |
|  |  |  |  |  |  |  |  |

OPA: Occupational physical activity. DPA: Domestic physical activity. LTPA: leisure-time physical activity

^a^ Model 1: model including age, family type and macroeconomic region and each domain of physical activity separately

^b^ Model 2: Model 1 plus general health status(SF-12 Physical Component Summary, SF-12 Mental Component Summary, Chronic morbidity index categories), health behaviours (smoking status and BMI categories) and educational qualification

^c^ Model 3: Model 2 plus adjustment for the other domains of physical activity

^d^ Coefficients based only on the 2004-2005 survey. Coefficients could not be calculated in the 1999-2000 survey due to lack of events in this category

^e^ Coefficients based only on the 1999-2000 survey. Coefficients could not be calculated in the 2004-2005 survey due to lack of events in this category

**Table 10: Gender-stratified association of domains of physical activity with all-cause mortality**

|  | **High education (post-diploma or degree, high school diploma)** | | |  | **Light education (middle school diploma, primary school diploma or less)** | | |
| --- | --- | --- | --- | --- | --- | --- | --- |
|  | **Model 1^a^** | **Model 2^b^** | **Model 3^c^** |  | **Model 1^a^** | **Model 2^b^** | **Model 3^c^** |
|  | **HR (95% CI)** | **HR (95% CI)** | **HR (95% CI)** |  | **HR (95% CI)** | **HR (95% CI)** | **HR (95% CI)** |
| **Men** |  |  |  |  |  |  |  |
| Light OPA | Ref | Ref | Ref |  | Ref | Ref | Ref |
| Moderate OPA | 1.05 (0.79-1.39) | 1.03 (0.80-1.32) | 1.04 (0.80-1.35) |  | 0.95 (0.75-1.20) | 0.98 (0.77-1.24) | 0.99 (0.78-1.26) |
| Heavy OPA | 0.81 (0.51-1.28) | 0.77 (0.49-1.22) | 0.76 (0.48-1.21) |  | 1.08 (0.79-1.48) | 1.07 (0.79-1.45) | 1.09 (0.78-1.51) |
| None DPA | Ref | Ref | Ref |  | Ref | Ref | Ref |
| Light DPA | 0.99 (0.76-1.29) | 0.96 (0.70-1.31) | 0.97 (0.67-1.39) |  | 1.06 (0.86-1.31) | 1.08 (0.85-1.37) | 1.11 (0.92-1.33) |
| Moderate DPA | 0.68 (0.50-0.91) | 0.66 (0.48-0.89) | 0.68 (0.50-0.92) |  | 0.70 (0.59-0.84) | 0.71 (0.60-0.84) | 0.72 (0.61-0.84) |
| Heavy DPA | 1.37 (0.86-2.16) | 1.30 (0.78-2.19) | 1.32 (0.74-2.36) |  | 0.84 (0.51-1.40) | 0.82 (0.48-1.40) | 0.80 (0.42-1.50) |
| None LTPA | Ref | Ref | Ref |  | Ref | Ref | Ref |
| Light LTPA | 0.74 (0.55-0.99) | 0.78 (0.58-1.06) | 0.80 (0.57-1.12) |  | 0.98 (0.78-1.23) | 1.00 (0.80-1.23) | 1.02 (0.86-1.22) |
| Moderate LTPA | 0.85 (0.45-1.59) | 0.95 (0.49-1.82) | 0.96 (0.49-1.90) |  | 0.82 (0.66-1.02) | 0.92 (0.73-1.15) | 0.96 (0.74-1.25) |
| Intense LTPA | 0.51 (0.27-0.97) | 0.63 (0.34-1.16) | 0.64 (0.34-1.21) |  | 0.81 (0.61-1.07) | 0.98 (0.75-1.27) | 1.00 (0.75-1.33) |
|  |  |  |  |  |  |  |  |
| **Women** |  |  |  |  |  |  |  |
| Light OPA | Ref | Ref | Ref |  | Ref | Ref | Ref |
| Moderate OPA | 1.15 (0.72-1.86) | 1.15 (0.74-1.79) | 1.14 (0.76-1.69) |  | 1.28 (0.88-1.88) | 1.24 (0.83-1.85) | 1.25 (0.83-1.86) |
| Heavy OPA | 0.43 (0.12-1.48) | 0.40 (0.11-1.43) | 0.41 (0.12-1.44) |  | 0.84 (0.48-1.49) | 0.74 (0.37-1.46) | 0.81 (0.45-1.45) |
| None DPA | 1.02 (0.30-3.45) ^d^ | 1.05 (0.30-3.67) ^d^ | 1.20 (0.33-4.34) ^d^ |  | 2.76 (1.11-6.85) | 2.37 (0.93-6.05) | 2.14 (0.85-5.34) |
| Light DPA | 0.77 (0.25-2.37) | 0.82 (0.26-2.62) | 0.83 (0.29-2.32) |  | 1.55 (0.27-8.92) | 1.56 (0.28-8.73) | 1.45 (0.29-7.32) |
| Moderate DPA | 0.93 (0.63-1.37) | 1.03 (0.70-1.51) | 1.02 (0.69-1.51) |  | 1.95 (0.80-4.77) | 2.06 (0.85-5.00) | 1.84 (0.83-4.07) |
| Heavy DPA | Ref | Ref | Ref |  | Ref | Ref | Ref |
| None LTPA | Ref | Ref | Ref |  | Ref | Ref | Ref |
| Light LTPA | 1.20 (0.77-1.87) | 1.27 (0.77-2.09) | 1.21 (0.75-1.94) |  | 0.62 (0.32-1.21) | 0.65 (0.36-1.16) | 0.64 (0.34-1.24) |
| Moderate LTPA | 1.24 (0.80-1.93) | 1.33 (0.86-2.04) | 1.26 (0.81-1.94) |  | 0.65 (0.44-0.96) | 0.70 (0.47-1.04) | 0.71 (0.48-1.07) |
| Intense LTPA | 1.05 (0.55-2.03) | 1.25 (0.64-2.44) | 1.21 (0.61-2.40) |  | 0.87 (0.42-1.77) | 0.95 (0.47-1.92) | 0.94 (0.47-1.88) |
|  |  |  |  |  |  |  |  |

OPA: Occupational physical activity. DPA: Domestic physical activity. LTPA: leisure-time physical activity

^a^ Model 1: model including age, family type and macroeconomic region and each domain of physical activity separately

^b^ Model 2: Model 1 plus general health status(SF-12 Physical Component Summary, SF-12 Mental Component Summary, Chronic morbidity index categories), health behaviours (smoking status and BMI categories) and educational qualification

^c^ Model 3: Model 2 plus adjustment for the other domains of physical activity

^d^ Coefficients based only on the 1999-2000 survey. Coefficients could not be calculated in the 2004-2005 survey due to lack of events in this category

**Table 11: Gender-stratified association of domains of physical activity with Coronary Heart Disease (CHD) events**

|  | **High education (post-diploma or degree, high school diploma)** | | |  | **Light education (middle school diploma, primary school diploma or less)** | | |
| --- | --- | --- | --- | --- | --- | --- | --- |
|  | **Model 1^a^** | **Model 2^b^** | **Model 3^c^** |  | **Model 1^a^** | **Model 2^b^** | **Model 3^c^** |
|  | **HR (95% CI)** | **HR (95% CI)** | **HR (95% CI)** |  | **HR (95% CI)** | **HR (95% CI)** | **HR (95% CI)** |
| **Men** |  |  |  |  |  |  |  |
| Light OPA | Ref | Ref | Ref |  | Ref | Ref | Ref |
| Moderate OPA | 1.04 (0.78-1.39) | 1.03 (0.75-1.42) | 1.05 (0.78-1.42) |  | 0.91 (0.68-1.22) | 0.97 (0.72-1.31) | 0.98 (0.73-1.32) |
| Heavy OPA | 1.08 (0.78-1.50) | 1.05 (0.76-1.45) | 1.07 (0.77-1.48) |  | 0.84 (0.69-1.02) | 0.87 (0.72-1.05) | 0.87 (0.72-1.04) |
| None DPA | Ref | Ref | Ref |  | Ref | Ref | Ref |
| Light DPA | 0.95 (0.79-1.14) | 0.94 (0.78-1.13) | 0.93 (0.77-1.11) |  | 1.07 (0.89-1.28) | 1.07 (0.90-1.28) | 1.06 (0.87-1.29) |
| Moderate DPA | 0.68 (0.52-0.88) | 0.68 (0.52-0.89) | 0.67 (0.51-0.87) |  | 0.91 (0.75-1.09) | 0.92 (0.76-1.10) | 0.91 (0.76-1.09) |
| Heavy DPA | 0.84 (0.53-1.35) | 0.84 (0.52-1.35) | 0.83 (0.51-1.34) |  | 1.03 (0.69-1.55) | 1.00 (0.67-1.48) | 1.01 (0.68-1.50) |
| None LTPA | Ref | Ref | Ref |  | Ref | Ref | Ref |
| Light LTPA | 1.12 (0.88-1.43) | 1.22 (0.96-1.54) | 1.27 (0.98-1.64) |  | 1.01 (0.79-1.28) | 1.02 (0.76-1.36) | 1.01 (0.75-1.37) |
| Moderate LTPA | 0.97 (0.73-1.30) | 1.10 (0.82-1.47) | 1.13 (0.85-1.51) |  | 0.95 (0.80-1.12) | 1.00 (0.86-1.17) | 0.99 (0.84-1.17) |
| Intense LTPA | 0.74 (0.53-1.03) | 0.93 (0.67-1.30) | 0.97 (0.69-1.35) |  | 0.75 (0.60-0.94) | 0.94 (0.73-1.22) | 0.93 (0.69-1.26) |
|  |  |  |  |  |  |  |  |
| **Women** |  |  |  |  |  |  |  |
| Light OPA | Ref | Ref | Ref |  | Ref | Ref | Ref |
| Moderate OPA | 0.92 (0.57-1.49) | 0.89 (0.54-1.48) | 0.88 (0.53-1.48) |  | 0.82 (0.58-1.17) | 0.83 (0.59-1.15) | 0.81 (0.58-1.13) |
| Heavy OPA | 0.53 (0.06-4.75) ^d^ | 0.45 (0.05-4.39) ^d^ | 0.42 (0.04-4.14) ^d^ |  | 0.80 (0.50-1.26) | 0.69 (0.38-1.25) | 0.67 (0.39-1.14) |
| None DPA | ^e^ | ^e^ | ^e^ |  | 1.53 (0.43-5.44) | 1.62 (0.46-5.77) | 1.51 (0.40-5.78) |
| Light DPA | 1.25 (0.39-3.94) | 1.21 (0.75-1.94) | 1.24 (0.33-4.62) |  | 0.48 (0.10-2.30) | 0.55 (0.12-2.52) | 0.51 (0.13-2.03) |
| Moderate DPA | 1.11 (0.68-1.81) | 1.26 (0.82-1.93) | 1.16 (0.71-1.88) |  | 0.89 (0.60-1.33) | 1.03 (0.71-1.51) | 1.01 (0.70-1.46) |
| Heavy DPA | Ref | Ref | Ref |  | Ref | Ref | Ref |
| None LTPA | Ref | Ref | Ref |  | Ref | Ref | Ref |
| Light LTPA | 1.22 (0.61-2.45) | 1.33 (0.76-2.31) | 1.31 (0.78-2.22) |  | 0.97 (0.61-1.54) | 0.97 (0.60-1.56) | 0.97 (0.60-1.58) |
| Moderate LTPA | 1.13 (0.65-1.95) | 1.15 (0.67-1.99) | 1.15 (0.65-2.06) |  | 0.92 (0.60-1.41) | 0.99 (0.63-1.55) | 0.98 (0.63-1.53) |
| Intense LTPA | 0.88 (0.35-2.21) | 0.98 (0.39-2.44) | 0.97 (0.39-2.40) |  | 0.70 (0.14-3.46) | 0.85 (0.13-5.65) | 0.82 (0.12-5.45) |
|  |  |  |  |  |  |  |  |

OPA: Occupational physical activity. DPA: Domestic physical activity. LTPA: leisure-time physical activity

^a^ Model 1: model including age, family type and macroeconomic region and each domain of physical activity separately

^b^ Model 2: Model 1 plus general health status(SF-12 Physical Component Summary, SF-12 Mental Component Summary, Chronic morbidity index categories), health behaviours (smoking status and BMI categories) and educational qualification

^c^ Model 3: Model 2 plus adjustment for the other domains of physical activity

^d^ Coefficients based only on the 2004-2005 survey. Coefficients could not be calculated in the 1999-2000 survey due to lack of events in this category

^e^ Coefficients could not be calculated because in both surveys due to lack of events in this category

**Table S12: Gender-stratified association of domains of physical activity with all-cause mortality, excluding participants with any chronic illness at baseline**

| After exclusion of 11,181 men and 8,064 women, there was a total of 556 deaths among men and 114 deaths among women | | | |
| --- | --- | --- | --- |
|  | **Model 1^a^** | **Model 2^b^** | **Model 3^c^** |
|  | **HR (95% CI)** | **HR (95% CI)** | **HR (95% CI)** |
| **Men** |  |  |  |
| Light OPA | Ref | Ref | Ref |
| Moderate OPA | 1.12 (0.92-1.36) | 0.99 (0.79-1.24) | 1.01 (0.81-1.27) |
| Heavy OPA | 1.27 (0.86-1.87) | 1.05 (0.57-1.93) | 1.06 (0.57-2.00) |
| None DPA | Ref | Ref | Ref |
| Light DPA | 1.28 (0.91-1.80) | 1.31 (0.90-1.93) | 1.32 (0.90-1.94) |
| Moderate DPA | 0.68 (0.52-0.89) | 0.70 (0.53-0.91) | 0.70 (0.53-0.92) |
| Heavy DPA | 0.81 (0.46-1.41) | 0.76 (0.48-1.21) | 0.78 (0.49-1.23) |
| None LTPA | Ref | Ref | Ref |
| Light LTPA | 0.95 (0.78-1.17) | 1.03 (0.84-1.26) | 1.03 (0.83-1.27) |
| Moderate LTPA | 0.84 (0.49-1.43) | 0.99 (0.57-1.70) | 1.03 (0.55-1.92) |
| Intense LTPA | 0.71 (0.43-1.19) | 0.88 (0.56-1.39) | 0.89 (0.52-1.51) |
|  |  |  |  |
| **Women** |  |  |  |
| Light OPA | Ref | Ref | Ref |
| Moderate OPA | 1.31 (0.89-1.95) | 1.44 (0.94-2.20) | 1.48 (0.97-2.27) |
| Heavy OPA | 1.04 (0.51-2.13) | 1.13 (0.50-2.55) | 1.26 (0.57-2.77) |
| None DPA | 1.07 (0.24-4.78) ^d^ | 1.06 (0.23-4.86) ^d^ | 1.05 (0.21-5.29) ^d^ |
| Light DPA | 1.50 (0.06-40.16) | 1.56 (0.06-42.16) | 1.66 (0.06-47.89) |
| Moderate DPA | 1.88 (0.39-9.12) | 1.96 (0.40-9.55) | 2.07 (0.41-10.54) |
| Heavy DPA | Ref | Ref | Ref |
| None LTPA | Ref | Ref | Ref |
| Light LTPA | 0.99 (0.63-1.54) | 0.99 (0.65-1.50) | 0.97 (0.64-1.48) |
| Moderate LTPA | 0.90 (0.55-1.47) | 0.93 (0.57-1.50) | 0.92 (0.56-1.51) |
| Intense LTPA | 1.34 (0.54-3.30) | 1.48 (0.57-3.86) | 1.51 (0.57-4.01) |
|  |  |  |  |

OPA: Occupational physical activity. DPA: Domestic physical activity. LTPA: leisure-time physical activity

^a^ Model 1: model including age, family type and macroeconomic region and each domain of physical activity separately

^b^ Model 2: Model 1 plus general health status(SF-12 Physical Component Summary, SF-12 Mental Component Summary, Chronic morbidity index categories), health behaviours (smoking status and BMI categories) and educational qualification

^c^ Model 3: Model 2 plus adjustment for the other domains of physical activity

^d^ Coefficients based only on the 1999-2000 survey. Coefficients could not be calculated in the 2004-2005 survey due to lack of events in this category

**Table S13: Gender-stratified association of domains of physical activity with Coronary Heart Disease (CHD) events, excluding participants with any chronic illness at baseline**

| After exclusion of 11,181 men and 8,064 women, there was a total of 640 CHD events among men and 50 CHD events among women | | | |
| --- | --- | --- | --- |
|  | **Model 1^a^** | **Model 2^b^** | **Model 3^c^** |
|  | **HR (95% CI)** | **HR (95% CI)** | **HR (95% CI)** |
| **Men** |  |  |  |
| Light OPA | Ref | Ref | Ref |
| Moderate OPA | 1.00 (0.77-1.30) | 0.93 (0.62-1.39) | 0.93 (0.63-1.39) |
| Heavy OPA | 1.02 (0.82-1.27) | 0.91 (0.69-1.20) | 0.90 (0.70-1.17) |
| None DPA | Ref | Ref | Ref |
| Light DPA | 0.93 (0.76-1.14) | 0.94 (0.76-1.16) | 0.93 (0.76-1.13) |
| Moderate DPA | 0.79 (0.65-0.96) | 0.80 (0.66-0.97) | 0.80 (0.66-0.96) |
| Heavy DPA | 0.75 (0.39-1.45) | 0.77 (0.39-1.50) | 0.76 (0.38-1.50) |
| None LTPA | Ref | Ref | Ref |
| Light LTPA | 1.16 (0.94-1.43) | 1.22 (0.98-1.51) | 1.24 (1.00-1.54) |
| Moderate LTPA | 0.89 (0.69-1.15) | 1.00 (0.77-1.30) | 1.02 (0.79-1.31) |
| Intense LTPA | 0.69 (0.53-0.89) | 0.84 (0.64-1.11) | 0.86 (0.65-1.13) |
|  |  |  |  |
| **Women** |  |  |  |
| Light OPA | Ref | Ref | Ref |
| Moderate OPA | 1.44 (0.79-2.63) | 1.11 (0.58-2.11) | 1.12 (0.59-2.10) |
| Heavy OPA | 1.48 (0.49-4.43) | 1.07 (0.34-3.35) | 1.02 (0.31-3.36) |
| None DPA | ^d^ | ^d^ | ^d^ |
| Light DPA | 0.32 (0.06-1.55) | 0.33 (0.06-1.64) | 0.30 (0.05-1.70) |
| Moderate DPA | 0.87 (0.41-1.81) | 0.86 (0.41-1.81) | 0.81 (0.35-1.89) |
| Heavy DPA | Ref | Ref | Ref |
| None LTPA | Ref | Ref | Ref |
| Light LTPA | 0.53 (0.29-0.97) | 0.59 (0.32-1.08) | 0.58 (0.31-1.08) |
| Moderate LTPA | 1.02 (0.49-2.09) | 1.15 (0.51-2.59) | 1.08 (0.50-2.31) |
| Intense LTPA | 1.08 (0.34-3.37) | 1.29 (0.40-4.19) | 1.28 (0.38-4.29) |
|  |  |  |  |

OPA: Occupational physical activity. DPA: Domestic physical activity. LTPA: leisure-time physical activity

^a^ Model 1: model including age, family type and macroeconomic region and each domain of physical activity separately

^b^ Model 2: Model 1 plus general health status(SF-12 Physical Component Summary, SF-12 Mental Component Summary, Chronic morbidity index categories), health behaviours (smoking status and BMI categories) and educational qualification

^c^ Model 3: Model 2 plus adjustment for the other domains of physical activity

^d^ Coefficients could not be calculated because in both surveys due to lack of events in this category

**Table S14: Gender-stratified association of domains of physical activity with all-cause mortality, excluding participants who had changed jobs within the past 5 years**

| After exclusion of 1,723 men and 1,746 women, there was a total of 1060 deaths among men and 291 deaths among women | | | |
| --- | --- | --- | --- |
|  | **Model 1^a^** | **Model 2^b^** | **Model 3^c^** |
|  | **HR (95% CI)** | **HR (95% CI)** | **HR (95% CI)** |
| **Men** |  |  |  |
| Light OPA | Ref | Ref | Ref |
| Moderate OPA | 1.12 (0.96-1.31) | 1.04 (0.85-1.28) | 1.05 (0.86-1.29) |
| Heavy OPA | 1.23 (1.01-1.51) | 1.01 (0.78-1.31) | 1.02 (0.78-1.35) |
| None DPA | Ref | Ref | Ref |
| Light DPA | 1.07 (0.88-1.30) | 1.10 (0.88-1.38) | 1.11 (0.90-1.38) |
| Moderate DPA | 0.71 (0.60-0.83) | 0.73 (0.61-0.86) | 0.74 (0.62-0.87) |
| Heavy DPA | 0.82 (0.37-1.82) | 0.81 (0.32-2.01) | 0.81 (0.31-2.12) |
| None LTPA | Ref | Ref | Ref |
| Light LTPA | 0.83 (0.66-1.04) | 0.88 (0.71-1.09) | 0.90 (0.75-1.08) |
| Moderate LTPA | 0.82 (0.60-1.12) | 0.98 (0.69-1.39) | 1.00 (0.68-1.47) |
| Intense LTPA | 0.61 (0.48-0.78) | 0.80 (0.61-1.04) | 0.80 (0.61-1.06) |
|  |  |  |  |
| **Women** |  |  |  |
| Light OPA | Ref | Ref | Ref |
| Moderate OPA | 1.12 (0.84-1.50) | 1.07 (0.82-1.41) | 1.08 (0.82-1.41) |
| Heavy OPA | 0.70 (0.35-1.41) | 0.63 (0.29-1.37) | 0.67 (0.33-1.36) |
| None DPA | 1.40 (0.69-2.83) | 1.39 (0.71-2.73) | 1.35 (0.69-2.65) |
| Light DPA | 1.02 (0.27-3.86) | 1.08 (0.30-3.88) | 1.00 (0.30-3.32) |
| Moderate DPA | 1.28 (0.85-1.91) | 1.37 (0.95-1.98) | 1.27 (0.97-1.66) |
| Heavy DPA | Ref | Ref | Ref |
| None LTPA | Ref | Ref | Ref |
| Light LTPA | 0.83 (0.54-1.28) | 0.86 (0.55-1.34) | 0.85 (0.52-1.37) |
| Moderate LTPA | 0.90 (0.72-1.12) | 0.97 (0.77-1.23) | 0.97 (0.76-1.23) |
| Intense LTPA | 0.98 (0.59-1.64) | 1.08 (0.65-1.82) | 1.07 (0.64-1.80) |
|  |  |  |  |

OPA: Occupational physical activity. DPA: Domestic physical activity. LTPA: leisure-time physical activity

^a^ Model 1: model including age, family type and macroeconomic region and each domain of physical activity separately

^b^ Model 2: Model 1 plus general health status(SF-12 Physical Component Summary, SF-12 Mental Component Summary, Chronic morbidity index categories), health behaviours (smoking status and BMI categories) and educational qualification

^c^ Model 3: Model 2 plus adjustment for the other domains of physical activity

**Table S15: Gender-stratified association of domains of physical activity with Coronary Heart Disease (CHD) events, excluding participants who had changed jobs within the past 5 years**

| After exclusion of 1,723 men and 1,746 women, there was a total of 1347 CHD events among men and 185 CHD events among women | | | |
| --- | --- | --- | --- |
|  | **Model 1^a^** | **Model 2^b^** | **Model 3^c^** |
|  | **HR (95% CI)** | **HR (95% CI)** | **HR (95% CI)** |
| **Men** |  |  |  |
| Light OPA | Ref | Ref | Ref |
| Moderate OPA | 1.06 (0.90-1.25) | 1.02 (0.78-1.32) | 1.03 (0.79-1.33) |
| Heavy OPA | 0.99 (0.84-1.16) | 0.92 (0.76-1.12) | 0.93 (0.78-1.10) |
| None DPA | Ref | Ref | Ref |
| Light DPA | 0.99 (0.84-1.17) | 1.00 (0.86-1.16) | 0.98 (0.80-1.19) |
| Moderate DPA | 0.79 (0.69-0.91) | 0.80 (0.70-0.92) | 0.79 (0.69-0.90) |
| Heavy DPA | 0.96 (0.66-1.38) | 0.93 (0.60-1.44) | 0.92 (0.59-1.46) |
| None LTPA | Ref | Ref | Ref |
| Light LTPA | 1.09 (0.86-1.39) | 1.13 (0.84-1.53) | 1.15 (0.83-1.58) |
| Moderate LTPA | 0.95 (0.79-1.15) | 1.09 (0.82-1.44) | 1.11 (0.82-1.50) |
| Intense LTPA | 0.73 (0.60-0.89) | 0.94 (0.77-1.15) | 0.95 (0.78-1.16) |
|  |  |  |  |
| **Women** |  |  |  |
| Light OPA | Ref | Ref | Ref |
| Moderate OPA | 1.00 (0.78-1.30) | 0.85 (0.65-1.10) | 0.85 (0.65-1.10) |
| Heavy OPA | 0.95 (0.58-1.55) | 0.68 (0.42-1.09) | 0.68 (0.42-1.10) |
| None DPA | 0.95 (0.30-3.04) | 1.09 (0.34-3.53) | 1.05 (0.32-3.44) |
| Light DPA | 0.79 (0.41-1.52) | 0.96 (0.50-1.84) | 0.88 (0.45-1.71) |
| Moderate DPA | 0.99 (0.73-1.34) | 1.18 (0.88-1.58) | 1.13 (0.86-1.49) |
| Heavy DPA | Ref | Ref | Ref |
| None LTPA | Ref | Ref | Ref |
| Light LTPA | 1.01 (0.73-1.41) | 1.09 (0.76-1.57) | 1.08 (0.74-1.58) |
| Moderate LTPA | 0.83 (0.56-1.22) | 0.94 (0.65-1.37) | 0.93 (0.63-1.38) |
| Intense LTPA | 0.55 (0.11-2.77) | 0.70 (0.11-4.48) | 0.70 (0.11-4.45) |
|  |  |  |  |

OPA: Occupational physical activity. DPA: Domestic physical activity. LTPA: leisure-time physical activity

^a^ Model 1: model including age, family type and macroeconomic region and each domain of physical activity separately

^b^ Model 2: Model 1 plus general health status(SF-12 Physical Component Summary, SF-12 Mental Component Summary, Chronic morbidity index categories), health behaviours (smoking status and BMI categories) and educational qualification

^c^ Model 3: Model 2 plus adjustment for the other domains of physical activity

**Appendix A: Physical activity questions in the Italian National Health Interview Surveys**

| **English** | | | | | | **OPA** | |
| --- | --- | --- | --- | --- | --- | --- | --- |
| **1999-2000 survey** | | | | | |  | |
| **Question (15.9), for employed only**: Is your work mostly characterised by physical activity: | | | | | |  | |
| 1. Light activity, most of the time is spent sitting | | | | | | Light | |
| 1. Modest activity, most of the time I am on my feet | | | | | | Moderate | |
| 1. Heavy activity, enough to make me sweat for around half the time at work | | | | | | Heavy | |
|  | | | | | |  | |
| **2004-2005 survey** | | | | | |  | |
| **Question (2.10), for employed only**: Is your work mostly characterised by physical activity? | | | | | |  | |
| 1. Light activity, most of the time is spent sitting | | | | | | Light | |
| 1. Moderate activity, most of the time I am on my feet | | | | | | Moderate | |
| 1. Heavy activity, enough to make me sweat | | | | | | Heavy | |
| **Italian** | | | | | | **OPA** | |
| **1999-2000 survey** | | | | | |  | |
| **Question (15.9), for employed only**: La sua attività lavorativa è prevalentemente  caratterizzata da attività fisica: | | | | | |  | |
| 1. Scarsa, la maggior parte del tempo è trascorsa stando seduti | | | | | | Light | |
| 1. Modesta, la maggior parte del tempo è trascorsa stando in piedi | | | | | | Moderate | |
| 1. Pesante, fino al punto di sudare per circa la metà delle ore di lavoro | | | | | | Heavy | |
|  | | | | | |  | |
| **2004-2005 survey** | | | | | |  | |
| **Question (2.10), for employed only**: La sua attività lavorativa è prevalentemente  caratterizzata da attività fisica: | | | | | |  | |
| 1. Scarsa, la maggior parte del tempo è trascorsa stando seduti | | | | | | Light | |
| 1. Modesta, la maggior parte del tempo è trascorsa stando in piedi | | | | | | Moderate | |
| 1. Pesante, fino al punto di sudare | | | | | | Heavy | |
|  | | | | | |  | |
| **English** | | | | | | **DPA** | |
| **1999-2000 survey** | | | | | |  | |
| **Question (15.6)**: Do you do housework? | | | | | |  | |
| 1. No | | | | | | None | |
| 1. Yes | | 🡪 if yes, go to question 15.7 | | | |  | |
| **Question (15.7)**: Is your domestic work mostly characterised by physical activity? | | | | | |  | |
| 1. Light activity | | | | | | Light | |
| 1. Moderate activity | | | | | | Moderate | |
| 1. Heavy activity, enough to make me sweat | | | | | | Heavy | |
|  | | | | | |  | |
| **2004-2005 survey** | | | | | |  | |
| **Question (17.2)**: How much time in hours and minutes do you spend on average per week doing domestic and family work (housework, grocery shopping, care of other family members, etc.)? | | | | | |  | |
| 1. No activity | | | | | | None | |
| Hours and minutes | | 🡪 go to question 17.3 | | | |  | |
| **Question (17.3)**: Is your work mostly characterised by physical activity? | | | | | |  | |
| 1. Yes, light activity | | | | | | Light | |
| 1. Yes, moderate activity | | | | | | Moderate | |
| 1. Yes, heavy activity, enough to make me sweat | | | | | | Heavy | |
| **Italian** | | | | | | **DPA** | |
| **1999-2000 survey** | | | | | |  | |
| **Question (15.6)**: Svolge lavoro domestico? | | | | | |  | |
| 1. No | | | | | | None | |
| 1. Sì | | 🡪 Se Sì, passare alla domanda 15.7 | | | |  | |
| **Question (15.7)**: Il suo lavoro domestico è prevalentemente  caratterizzato da attività fisica? | | | | | |  | |
| 1. Scarsa | | | | | | Light | |
| 1. Modesta | | | | | | Moderate | |
| 1. Pesante, fino al punto di sudare un po’ | | | | | | Heavy | |
|  | | | | | |  | |
| **2004-2005 survey** | | | | | |  | |
| **Question (17.2)**: Quanto tempo in ore e minuti dedica mediamente alla settimana al lavoro domestico e familiare (faccende di casa, fare la spesa, cura di altri componenti): | | | | | |  | |
| 1. Non svolge attività | | | | | | None | |
| N. ore N. minuti | | 🡪 passare alla domanda 17.3 | | | |  | |
| **Question (17.3)**: Il suo lavoro domestico e familiare è prevalentemente caratterizzato da attività fisica? | | | | | |  | |
| 1. SÌ, scarsa | | | | | | Light | |
| 1. SÌ, scarsa | | | | | | Moderate | |
| 1. SÌ, scarsa | | | | | | Heavy | |
|  | | | | | |  | |
| **English** | | | | | | | **LTPA** |
| **1999-2000 survey** | | | | | | |  |
| **Question (15.1)**: In the last 12 months during your leisure time have you usually practised one or more sports or physical activities which required intensive training (competitive sports, intense gym exercising, etc.)? | | | | | | |  |
| 1. No | | | | 🡪if not, go to question 15.2 | | |  |
| 1. Yes, more than 4 hours a week | | | | | | | Intense |
| 1. Yes, 4 hours a week | | | | | | | Intense |
| 1. Yes, less than 4 hours a week | | | | | | | Intense |
| **Question (15.2)**: In the last 12 months during your leisure time have you regularly practised one or more sports or physical activities until to make you sweat (jogging, light gym exercising, cycling, gardening, etc.)? | | | | | | |  |
| 1. No | | | | 🡪if not, go to question 15.3 | | |  |
| 1. Yes, more than 4 hours a week | | | | | | | Moderate |
| 1. Yes, 4 hours a week | | | | | | | Moderate |
| 1. Yes, less than 4 hours a week | | | | | | | Moderate |
| **Question (15.3)**: Do you usually do light physical activity (walking for at least 1 km, climbing stairs, etc.) | | | | | | |  |
| 1. No | | | | | | | None |
| 1. Yes, more than 4 hours a week | | | | | | | Light |
| 1. Yes, 4 hours a week | | | | | | | Light |
| 1. Yes, less than 4 hours a week | | | | | | | Light |
| **2004-2005 survey** | | | | | **Minutes of LTPA** | |  |
| **Question (17.1)**: In your free time do you do at least once a week (read each answer and complete row by row): | | | | |  | |  |
| **A. Heavy physical activity (competitive sports, intense gym exercising, etc.)** | | | | |  | |  |
| 1. No | | | | | 0’ | |  |
| 2. Yes 🡪 | How many days per week? 🡪 | | How many minutes on average, each time? | |  | |  |
|  | Number of days | | 1. Less than 20 minutes | | N days × 10’ | | Intense |
|  |  | | 2. 20 minutes or more  🡪 Number of minutes | | N days × N’ | | Intense |
| 1. **B. Moderate physical activity (light** **gym exercising, moderate speed bike rides, etc.)** | | | | |  | |  |
| 1. No | | | | | 0’ | |  |
| 2. Yes 🡪 | How many days per week? 🡪 | | How many minutes on average, each time? | |  | |  |
|  | Number of days | | 1. Less than 20 minutes | | N days × 10’ | | Moderate^a^ |
|  |  | | 2. 20 minutes or more  🡪 Number of minutes | | N days × N’ | | Moderate^a^ |
| 1. **C. Light physical activity (walking for at least 1 km, gentle exercising, etc.)** | | | | |  | |  |
| 1. No | | | | | 0’ | | None^b^ |
| 2. Yes 🡪 | How many days per week? 🡪 | | How many minutes on average, each time? | |  | |  |
|  | Number of days | | 1. Less than 20 minutes | | N days × 10’ | | Light^c^ |
|  |  | | 2. 20 minutes or more  🡪 Number of minutes | | N days × N’ | | Light^c^ |
| ^a^ if subject answers “1. No” to point “A. Heavy physical activity”  ^b^ if subject answers “1. No” to all three points “A. Heavy physical activity”, “B. Moderate physical activity” and “C. Light physical activity”  ^c^ if subject answers “1. No” to both points “A. Heavy physical activity” and “B. Moderate physical activity” | | | | | | | |
| **Italian** | | | | | | | **LTPA** |
| **1999-2000 survey** | | | | | | |  |
| - 1. **Question (15.1)**: Nel suo tempo libero ha praticato negli ultimi 12 mesi con carattere di continuità una o più attività fisica o sportiva che ha richiesto ALLENAMENTI INTENSIVI (sport agonistici e non, palestra ecc..)? | | | | | | |  |
| 1. No | | | | 🡪Se no, passare alla domanda 15.2 | | |  |
| 1. SI, oltre 4ore a settimana | | | | | | | Intense |
| 1. SI, 4ore a settimana | | | | | | | Intense |
| 1. SI, meno di 4 ore a settimana | | | | | | | Intense |
| **Question (15.2)**: Nel suo tempo libero ha praticato negli ultimi 12 mesi regolarmente una o più attività fisica o motoria FINO AL PUNTO DI SUDARE UN PO' (jogging, palestra, passeggiate in bicicletta, giardinaggio, ecc..)? | | | | | | |  |
| 1. No | | | | 🡪Se no, passare alla domanda 15.3 | | |  |
| 1. SI, oltre 4ore a settimana | | | | | | | Moderate |
| 1. SI, 4ore a settimana | | | | | | | Moderate |
| 1. SI, meno di 4 ore a settimana | | | | | | | Moderate |
| **Question (15.3)**: Svolge abitualmente una attività fisica e motoria LEGGERA (passeggiate a piedi per almeno un chilometro, salire le scale ecc..)? | | | | | | |  |
| 1. No | | | | | | | None |
| 1. SI, oltre 4ore a settimana | | | | | | | Light |
| 1. SI, 4ore a settimana | | | | | | | Light |
| 1. SI, meno di 4 ore a settimana | | | | | | | Light |
| **2004-2005 survey** | | | | | **Minutes of LTPA** | |  |
| **Question (17.1)**: Nel suo tempo libero, pratica, almeno una volta la settimana (Leggere tutte le risposte e compilare riga per riga) | | | | |  | |  |
| **A.**  attività sportiva con rilevante sforzo fisico (sport agonistici e non, palestra, ciclismo, jogging ecc..)? | | | | |  | |  |
| 1. No | | | | | 0’ | |  |
| 2. Sì 🡪 | Per quanti giorni la settimana | | Per quanti minuti ogni volta in media | |  | |  |
|  | N | | 1. Meno di 20 minuti | | N days × 10’ | | Intense |
|  |  | | 2. 20 minuti e più 🡪 specificare in minuti | | N days × N’ | | Intense |
| 1. **B.**  attività fisica moderata, cioè fino al punto di sudare un po’ (palestra, passeggiate in bicicletta a velocità moderata, ecc..)? | | | | |  | |  |
| 1. No | | | | | 0’ | |  |
| 2. Sì 🡪 | Per quanti giorni la settimana | | Per quanti minuti ogni volta in media | |  | |  |
|  | N | | 1. Meno di 20 minuti | | N days × 10’ | | Moderate^a^ |
|  |  | | 2. 20 minuti e più 🡪 specificare in minuti | | N days × N’ | | Moderate^a^ |
| 1. **C.**  attività fisica leggera (passeggiate a piedi per almeno 1 km, ginnastica dolce, ecc.)? | | | | |  | |  |
| 1. No | | | | | 0’ | | None^b^ |
| 2. Sì 🡪 | Per quanti giorni la settimana | | Per quanti minuti ogni volta in media | |  | |  |
|  | N | | 1. Meno di 20 minuti | | N days × 10’ | | Light^c^ |
|  |  | | 2. 20 minuti e più 🡪 specificare in minuti | | N days × N’ | | Light^c^ |
| ^a^ if subject answers “1. No” to point “A. Heavy physical activity”  ^b^ if subject answers “1. No” to all three points “A. Heavy physical activity”, “B. Moderate physical activity” and “C. Light physical activity”  ^c^ if subject answers “1. No” to both points “A. Heavy physical activity” and “B. Moderate physical activity” | | | | | | | |

**Appendix B: STROBE Statement—checklist of items that should be included in reports of observational studies**

|  | Item No | Recommendation | Page  No |
| --- | --- | --- | --- |
| **Title and abstract** | 1 | (*a*) Indicate the study’s design with a commonly used term in the title or the abstract | 1 |
|  |  | (*b*) Provide in the abstract an informative and balanced summary of what was done and what was found | 4 |
| Introduction | | | |
| Background/rationale | 2 | Explain the scientific background and rationale for the investigation being reported | 5-7 |
| Objectives | 3 | State specific objectives, including any prespecified hypotheses | 7 |
| Methods | | | |
| Study design | 4 | Present key elements of study design early in the paper | 7-8 |
| Setting | 5 | Describe the setting, locations, and relevant dates, including periods of recruitment, exposure, follow-up, and data collection | 7-8 |
| Participants | 6 | (*a*) *Cohort study*—Give the eligibility criteria, and the sources and methods of selection of participants. Describe methods of follow-up  *Case-control study*—Give the eligibility criteria, and the sources and methods of case ascertainment and control selection. Give the rationale for the choice of cases and controls  *Cross-sectional study*—Give the eligibility criteria, and the sources and methods of selection of participants | 7-8 |
|  |  | (*b*) *Cohort study*—For matched studies, give matching criteria and number of exposed and unexposed  *Case-control study*—For matched studies, give matching criteria and the number of controls per case | NA |
| Variables | 7 | Clearly define all outcomes, exposures, predictors, potential confounders, and effect modifiers. Give diagnostic criteria, if applicable | 9-10 |
| Data sources/ measurement | 8* | For each variable of interest, give sources of data and details of methods of assessment (measurement). Describe comparability of assessment methods if there is more than one group | 9-10 |
| Bias | 9 | Describe any efforts to address potential sources of bias | 8-10 |
| Study size | 10 | Explain how the study size was arrived at | 8 |
| Quantitative variables | 11 | Explain how quantitative variables were handled in the analyses. If applicable, describe which groupings were chosen and why | 9-10 |
| Statistical methods | 12 | (*a*) Describe all statistical methods, including those used to control for confounding | 10-11 |
|  |  | (*b*) Describe any methods used to examine subgroups and interactions | 10-11 |
|  |  | (*c*) Explain how missing data were addressed |  |
|  |  | (*d*) *Cohort study*—If applicable, explain how loss to follow-up was addressed  *Case-control study*—If applicable, explain how matching of cases and controls was addressed  *Cross-sectional study*—If applicable, describe analytical methods taking account of sampling strategy |  |
|  |  | (*e*) Describe any sensitivity analyses | 11 |

| Results | | | | |
| --- | --- | --- | --- | --- |
| Participants | 13* | (a) Report numbers of individuals at each stage of study—eg numbers potentially eligible, examined for eligibility, confirmed eligible, included in the study, completing follow-up, and analysed | 7 |  |
|  |  | (b) Give reasons for non-participation at each stage |  |  |
|  |  | (c) Consider use of a flow diagram |  |  |
| Descriptive data | 14* | (a) Give characteristics of study participants (eg demographic, clinical, social) and information on exposures and potential confounders | 16 |  |
|  |  | (b) Indicate number of participants with missing data for each variable of interest | 16 |  |
|  |  | (c) *Cohort study*—Summarise follow-up time (eg, average and total amount) | Table S3 |  |
| Outcome data | 15* | *Cohort study*—Report numbers of outcome events or summary measures over time | Table S3 |  |
|  |  | *Case-control study—*Report numbers in each exposure category, or summary measures of exposure |  |  |
|  |  | *Cross-sectional study—*Report numbers of outcome events or summary measures |  |  |
| Main results | 16 | (*a*) Give unadjusted estimates and, if applicable, confounder-adjusted estimates and their precision (eg, 95% confidence interval). Make clear which confounders were adjusted for and why they were included | 11-12 |  |
|  |  | (*b*) Report category boundaries when continuous variables were categorized | NA |  |
|  |  | (*c*) If relevant, consider translating estimates of relative risk into absolute risk for a meaningful time period | NA |  |
| Other analyses | 17 | Report other analyses done—eg analyses of subgroups and interactions, and sensitivity analyses | 12 |  |
| Discussion | | | | |
| Key results | 18 | Summarise key results with reference to study objectives | 13 |  |
| Limitations | 19 | Discuss limitations of the study, taking into account sources of potential bias or imprecision. Discuss both direction and magnitude of any potential bias | 12-15 |  |
| Interpretation | 20 | Give a cautious overall interpretation of results considering objectives, limitations, multiplicity of analyses, results from similar studies, and other relevant evidence | 15 |  |
| Generalisability | 21 | Discuss the generalisability (external validity) of the study results | 15 |  |
| Other information | | | | |
| Funding | 22 | Give the source of funding and the role of the funders for the present study and, if applicable, for the original study on which the present article is based | 2 |  |

*Give information separately for cases and controls in case-control studies and, if applicable, for exposed and unexposed groups in cohort and cross-sectional studies.

**Note:** An Explanation and Elaboration article discusses each checklist item and gives methodological background and published examples of transparent reporting. The STROBE checklist is best used in conjunction with this article (freely available on the Web sites of PLoS Medicine at http://www.plosmedicine.org/, Annals of Internal Medicine at http://www.annals.org/, and Epidemiology at http://www.epidem.com/). Information on the STROBE Initiative is available at www.strobe-statement.org.
